# A Local Learning Rule for Independent Component Analysis

**DOI:** 10.1038/srep28073

**Published:** 2016-06-21

**Authors:** Takuya Isomura, Taro Toyoizumi

**Affiliations:** 1 RIKEN Brain Science Institute, 2-1 Hirosawa, Wako, Saitama 351-0198, Japan; 2 Department of Human and Engineered Environmental Studies, Graduate School of Frontier Sciences, The University of Tokyo, 7-3-1 Hongo, Bunkyo-ku, Tokyo 113-8656, Japan; 3 Research Fellow of Japan Society for the Promotion of Science (JSPS), 5-3-1 Kojimachi, Chiyoda-ku, Tokyo 102-0083, Japan

## Abstract

Humans can separately recognize independent sources when they sense their superposition. This decomposition is mathematically formulated as independent component analysis (ICA). While a few biologically plausible learning rules, so-called local learning rules, have been proposed to achieve ICA, their performance varies depending on the parameters characterizing the mixed signals. Here, we propose a new learning rule that is both easy to implement and reliable. Both mathematical and numerical analyses confirm that the proposed rule outperforms other local learning rules over a wide range of parameters. Notably, unlike other rules, the proposed rule can separate independent sources without any preprocessing, even if the number of sources is unknown. The successful performance of the proposed rule is then demonstrated using natural images and movies. We discuss the implications of this finding for our understanding of neuronal information processing and its promising applications to neuromorphic engineering.

One remarkable power of the brain is that it can rapidly identify “objects” from their mixtures. The visual cortex can rapidly identify multiple objects in natural scenes^1^, and the auditory cortex can recognize a talker in a noisy social environment, a phenomenon known as the cocktail party effect^2^,^3^,^4^. The problem of separating sensory sources while blind to how they are mixed is termed blind source separation (BSS), which is believed essential for various cognitive tasks^5^,^6^,^7^,^8^. Hence, how BSS is performed in the brain can provide a key insight into the way the brain processes sensory information.

Independent component analysis (ICA)^9^ is a mathematical model of BSS, where an observer receives linear mixtures of independent sources as inputs and determines the transformation back into their original sources without knowing how they are mixed in the first place. Notably, explicit supervision of which stimulus features belong to what sources is not required to perform ICA. A learner can spontaneously develop the ability to separate independent components only based on stimulus statistics–a concept developed in machine learning as unsupervised learning^10^,^11^,^12^. Several such ICA algorithms (also called learning rules) have been proposed, including those that are based on the information maximization principle^13^,^14^,^15^,^16^ and the non-Gaussianity of signals^17^. While these learning rules have been successfully used in many engineering applications^18^, their neural implementation is not straightforward because each neuron needs to know the information of other unconnected neurons under these rules^16^. Therefore, these learning rules are called *non-local*.

There are a few ICA learning rules that use only the local information available in each neuron and are thus biologically more plausible^19^,^20^,^21^. However, a drawback is that they do not always converge to a desirable solution. A biologically plausible learning rule with reliable performance remains under open investigation.

Here, we proposed a new biologically plausible local learning rule for ICA. First, we propose an extended Hebbian learning rule^22^, where changes in synaptic strength are gated by a global error signal summed over a local neural population. We show that this rule can be derived as a gradient descent rule of a cost function that approximates the mutual information between output neurons. Second, we theoretically analyze the stability and uniqueness of its solution. Third, we compare it with other ICA rules and demonstrate that, unlike conventional local rules, the proposed rule reliably converges to an ideal solution over a wide range of mixing matrices, source distributions, and source time scales. Finally, we indicate its promising applications in neuromorphic engineering using natural images and movies.

## Results

### A novel local ICA learning rule

**Figure 1:**
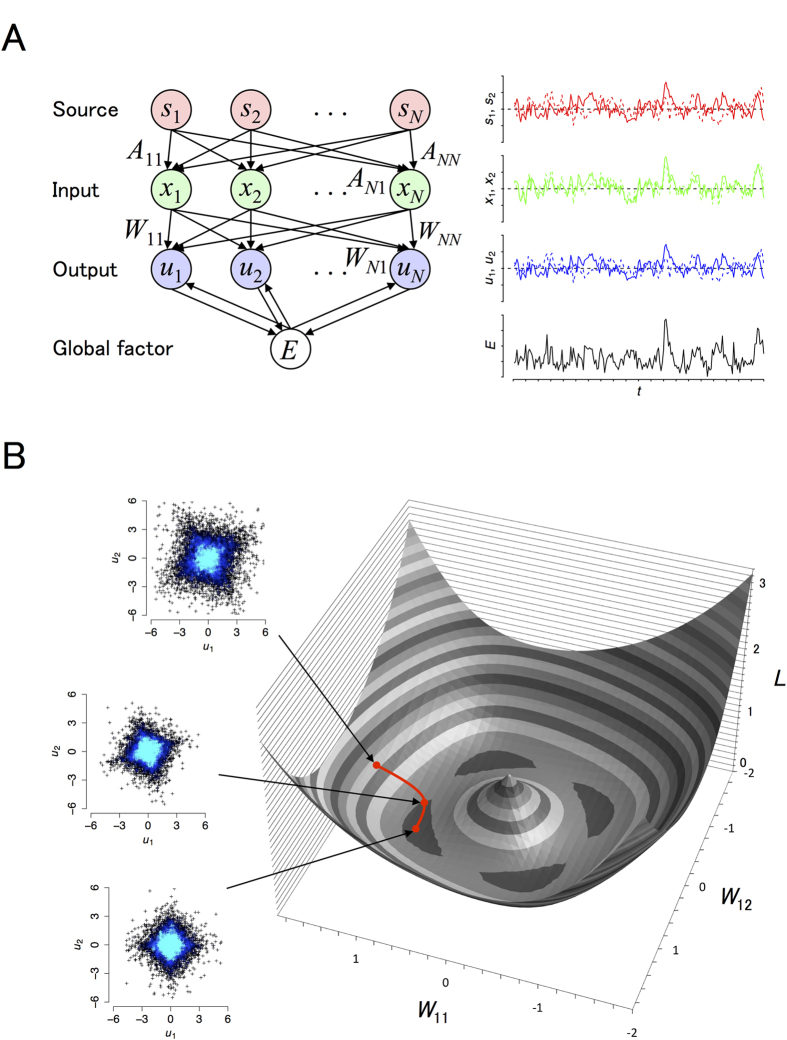
Schematic image of the model setup and results of the proposed learning rule. **(A)** Left: Schematic image of the model. The input **x** to the neural network is a linear mixture of independent sources **s**, i.e., **x** = *A***s**, where *A* is a mixing matrix. The neural network linearly sums the input and produces the output **u** = *W***x**, where *W* is a synaptic strength matrix. The goal is to learn the *W* ∝ *A*^−1^ (or its row permutations and signflips) for which the outputs become independent. To this end, a global signal *E* is computed based on the outputs of individual neurons and gate activity-dependent changes in *W* during learning. Right: Time traces of **s**, **x**, **u**, and *E*. **(B)** A dynamic trajectory of the synaptic strength matrix while the network learns to separate independent sources. The learning rule is formulated as a gradient descent algorithm of a cost function *L*, whose landscape is depicted as a function of synaptic strength parameters (*W*_11_, *W*_12_). Note that in order to graphically illustrate the results in this three-dimensional plot, we used a two-dimensional rotation matrix with angle 6/*π* as the mixing matrix *A* and restricted *W* as a rotation and scaling matrix (*W*_11_, *W*_12_; −*W*_12_, *W*_11_). The red trajectory displays how the gradient descent algorithm reduces the cost function *L* by adjusting (*W*_11_, *W*_12_). The three inset panels display the distributions of the network output (*u*_1_, *u*_2_) during the course of the learning. Each point represents sampled outputs and the brightness of the blue color represents probability density. Top: The outputs are not independent at the initial condition (*W*_11_, *W*_12_) = (1.5, 0). Middle: The distribution of the outputs is rotated during learning. Bottom: The network outputs become independent at the final state (*W*_11_, *W*_12_) = (cos 6/*π*, sin 6/*π*). The two sources are drawn independently from the same Laplace distribution (see Methods). Note that a MATLAB source code of the EGHR is appended as Supplementary Source Code 1.

The basic problem is to recover a vector of unobserved independent sources **s** from its linear mixture **x** = *A***s** without knowing the mixing matrix *A*. We assume that the independent sources are distributed according to an identical probability distribution, i.e., Prob(**s**) = ∏_*i*_ *p*_0_(*s*_*i*_). Note that sources must follow a non-Gaussian distribution for ICA to be successful (in Section 7.5 in ref. 9). In this work, we consider a network of *N* neurons that learn to separate independent sources (Fig. 1A). The output of these neurons is computed by **u** = *W***x**, where **x** is the activity of the input neurons and *W* is a matrix of synaptic strengths from the input neurons. Note that each element of **s**, **x**, and **u** may take a positive or negative value here, as they represent a relative rather than absolute activity level. The goal is to find a synaptic strength matrix that produces independent output. One solution is *W* = *A*^−1^, whereby **u** = **s** is achieved, but any additional permutations and signflips of the output elements also give a solution. We collectively call them ICA solutions. Except when we consider the undercomplete condition later, we assume **s**, **x**, and **u** are *N*-dimensional column vectors.

Conventional ICA rules often modify synaptic strength depending on a product of pre- and post-synaptic activity. We called them Hebbian^11^,^22^ rules in a broad sense. Neurons tend to receive a correlated group of inputs under a Hebbian rule. In addition, previous local ICA rules use lateral inhibition to decorrelate the activities of output neurons^19^,^20^,^21^. These mechanisms help neurons to represent separate independent sources. However, modification of neural activity by lateral inhibition is not the only way a neuron influences synaptic plasticity of other neurons. A number of experimental studies have reported that a third-factor, apart from pre- and post-synaptic activity, can play an essential role in modulating the outcome of Hebbian plasticity. For example, GABA^23^,^24^, dopamine^25^,^26^,^27^, noradrenalin^28^,^29^, and D-serin^30^ are known to modulate Hebbian plasticity. Therefore, local ICA computation may be possible without the need for direct lateral inhibition, if such a third factor monitors the overall state of the neurons and adequately modulates Hebbian plasticity.

In this study, we proposed a novel local learning rule for ICA that extends a Hebbian learning rule by a time varying learning rate, based on a global error signal. We call this the error-gated Hebbian rule (EGHR), expressed as follows:

EGHR


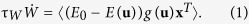


Here, the *g*(*u*_*i*_)*x*_*j*_ term is a standard Hebbian term, commonly included in many ICA rules (c.f., Equations 2, 3, 4, 5, 6), where *g*(*u*_*i*_) = −dlog *p*_0_(*u*_*i*_)/d*u*_*i*_ describes a postsynaptic factor of neuron *i* and *x*_*j*_ describes a presynaptic factor of neuron *j*. Further, 〈•〉 describes an expectation over the ensemble of **x**, the dot over *W* denotes a temporal derivative, and τ_*W*_ is a learning time-constant. In this rule, this Hebbian term is gated by a global error signal *E*_0_ − *E*(**u**) composed of constant *E*_0_ and *E*(**u**) = −∑_*i*_ log *p*_0_(*u*_*i*_). The term *E*(**u**) describes the surprise^31^ of observing output **u** under the assumption that the distribution of output is ∏_*i*_ *p*_0_(*u*_*i*_), which is achieved after successful learning. Accordingly, the EGHR operation switches with the error signal; the EGHR facilitates a current activity pattern by inducing Hebbian change if *E*_0_ > *E*(**u**). In contrast, it suppresses the pattern by inducing anti-Hebbian change if *E*_0_ < *E*(**u**). In this way, the *E*GHR maintains the error *E*_0_ − *E*(**u**) close to zero.

Indeed, it is easy to demonstrate that the EGHR is a gradient descent rule that minimizes a cost function *L* = 〈(*E*(**u**) − *E*_0_)^2^/2〉 (see Methods). Hence, the basic strategy behind the EGHR is to reduce the fluctuations of *E*(**u**) while maintaining its average close to *E*_0_. Independence between outputs (as opposed to highly correlated outputs) helps to keep the fluctuations of *E*(**u**) small. In addition, *E*_0_ (>−*N* log *p*_0_(0)) prevents *W* from converging to zero, avoiding the trivial solution of **u** = **0**. It turns out that *L* approximates the common cost function of the Bell-Sejnowski and Amari rules^13^,^14^,^15^ if *W* is near an ICA solution (see Methods and S2.2). Despite this similarity, the EGHR is more biologically plausible than the Bell-Sejnowski and Amari rules because its synaptic changes are based on the local information^16^ available at each synapse (see the next section). Note that the computational complexity required by the EGHR is of an *N*^2^-order, *O*(*N*^2^), in a serial implementation, but is *O*(*N*) in a parallel implementation such as using a neuromorphic hardware.

The simple EGHR can straightforwardly perform ICA, as we illustrate using an example with two independent sources obeying a Laplace distribution (Fig. 1B). Figure 1B left shows a typical outcome of the EGHR, where initially non-independent outputs become independent along a gradient descent path of the cost function *L* (Fig. 1B right; see Methods). We show in separate simulations that the outcome of this rule is robust to the number of independent sources, distribution from which the sources are generated, and deviations of *E*(**u**) and *g*(**u**) from the above definitions due to the unknown form of the source distribution *p*_0_ (Supplementary Movie 1 and Supplementary Figs S1–3; see also Supplementary Movie 2 for an example with natural scenes). In addition, employing detailed theoretical analyses, we show (Methods and Supplementary information S2.2–4): (1) a mathematical condition under which the ICA solutions are stable fixed points of the EGHR, (2) that the ICA solutions are unique solutions of the EGHR if the source distribution is nearly Gaussian, and (3) the robustness of EGHR solutions to the choice of *E*_0_.

### Comparison of ICA rules

In this section, we compare the EGHR with five conventional ICA rules:

1. EGHR:


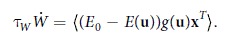


2. Bell-Sejnowski^13^,^14^:


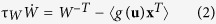


3. Amari^15^:


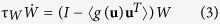


4. Cichocki^21^:


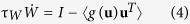


5. Linsker^20^:


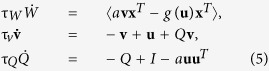


6. Foldiak^19^:


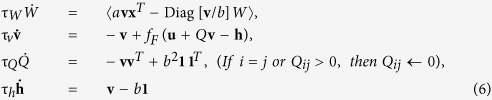


In these rules, again **x** = *A***s** is the input to each model and **u** = *W***x** is its output. The *g*(**u**)**x**^*T*^ (or *g*(**u**)**u**^*T*^*W*) term is common across many ICA rules. In addition to the dynamics of *W*, the Linsker and Foldiak rules assume dynamic updates of neural state **v** and lateral connections *Q*. The Foldiak rule additionally assumes an adaptive threshold **h**. Note that τ_•_ describes the time constant of dynamical variable •, *I* is the *N* × *N* identity matrix, **1** is an *N*-dimensional vector of ones, *a* and *b* are constant parameters that we vary in the following, and *f*_*F*_(•) is a nonlinear function. While *a* = 1 in the original Foldiak rule, tuning *a* is important in some cases, as we describe below (see Table 1 for parameter values).

Importantly, the Bell-Sejnowki and Amari rules are so called non-local learning rules^16^ because updating synaptic strength *W*_*ij*_ requires the information of remote synapses such as *W*_*kl*_, where *i* ≠ *k* or *j* ≠ *l*. On the other hand, the Cichocki, Linsker, and Foldiak rules are so called local learning rules because each synapse is updated based on quantities available there. Note that while the Cichocki rule does not require lateral connections to modify neural activity, they may be required to signal the activity of one neuron to another to achieve a local implementation of the learning rule.

Notably, the Linsker and Foldiak rules are more involved than the others because they need to learn a few sets of dynamical variables in addition to synaptic weight matrix *W*. In order for these rules to work, the time constants of **s**, **v**, *Q*, and *W* must satisfy τ_*v*_ < τ_*s*_ ≪ τ_*Q*_ < τ_*W*_. This means that the neuronal time constant must be faster than the input time constant and learning needs to be a couple of orders of magnitude slower than the neuronal time constant for these rules. Hence, for these learning rules, we need to introduce slowly time-varying sources. Specifically, we model dynamical sources (*i* = 1, …, *N*) according to


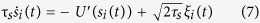


where *U′* (*s*_*i*_) is a derivative of a potential function, *ξ*_*i*_(*t*) is a white Gaussian random variable of unit variance and τ_*s*_ is the time constant of the sources. The marginal distribution of each source is given in terms of the potential function by *p*_0_(*s*_*i*_(*t*)) ∝ exp(−*U*(*s*_*i*_(*t*))). Hence, each source is distributed according to a Laplace distribution with zero mean and a variance of one if the potential function is *U*_*L*_(*s*_*i*_) = 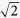
|*s*_*i*_| and a uniform distribution with zero mean and a variance of one if the potential function is *U*_*U*_(*s*_*i*_) = 0 for |*s*_*i*_| ≤ 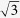 and a large positive constant for |*s*_*i*_| > 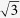.

**Figure 2:**
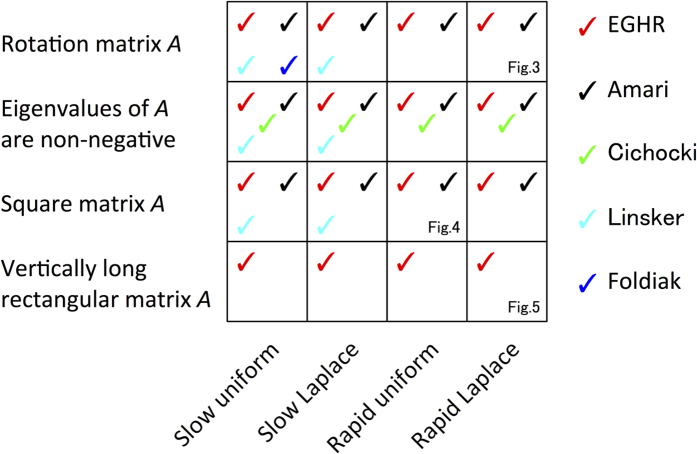
Comparison of ICA rules. Rows and columns describe the features of mixing matrix *A* and sources **s**, respectively. Each tick mark indicates that the rule can always perform ICA under the corresponding condition according to our analytical (see Methods and Supplementary Note S2) and numerical (see Figs 3, 4, 5 and S1–3) analyses. The EGHR can perform ICA under all listed conditions (see Methods, S2.2–4, Figs 3, 4, 5, and S1–3). Previous rules all fail to perform ICA under the undercomplete condition with vertically long rectangular matrix *A* (see S2.8 and Fig. 5). Under the Linsker rule, the output can converge to zero or diverge to infinity if the sources fluctuate more rapidly than the neuronal time constant (see S2.6.2). The Cichocki rule fails if all eigenvalues of *A* are negative and can fail depending on the initial synaptic strengths if some eigenvalues are negative (see S2.7.2 and Figs 3 and 4). The Foldiak rule works only if *A* is proportional to a rotation matrix (see S2.6.3 and Fig. 4). In addition, the Foldiak rule has multiple spurious stable solutions if the sources obey a Laplace distribution (see Fig. 3). Note that the properties of the Bell-Sejnowski rule are the same as those of the Amari rule.

**Figure 3:**
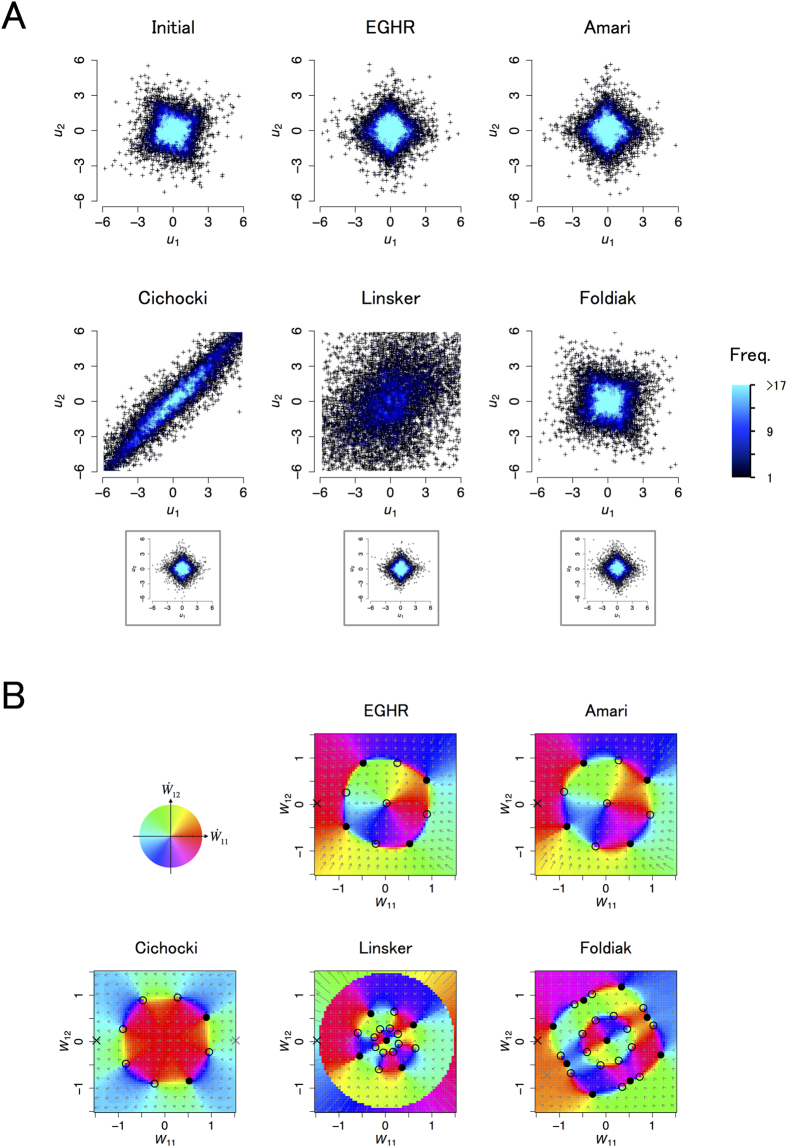
Results of ICA with a rotation mixing matrix and Laplace source distribution. **(A)** Initial and final distribution of **u** = (*u*_1_, *u*_2_) for each rule. Top left: Initial distribution of outputs common to all rules. Other panels: Final distribution of outputs for each rule. Horizontal and vertical axes respectively represent *u*_1_ and *u*_2_. Panels show samples of output signals pooled over the first or last 10^4^ steps. **(B)** Velocity map of each ICA rule. Horizontal and vertical axes respectively indicate *W*_11_ and *W*_12_, where synaptic strength matrix *W* = (*W*_11_, *W*_12_; −*W*_12_, *W*_11_). The direction of the arrow and color at each location represent the direction of the change of synaptic strengths, (*W*_11_, *W*_12_), and the length of the arrow represents the magnitude of the change. ICA solutions are located at (*W*_11_, *W*_12_) = (cos *θ*, sin *θ*) for *θ* = *π*/6, 2*π*/3, 7*π*/6, and 5*π*/3. Top left: The color scale. Other panels: Velocity maps for the EGHR, Amari, Linsker, Cichocki, and Foldiak rules, respectively. The time constant of the sources was *τ*_*s*_ = 50. The neuronal time constant for the Linsker and Foldiak rules was *τ*_*v*_ = 10. The filled and open circles respectively indicate stable and unstable equilibrium points. The black and gray cross marks respectively indicate the common initial condition used in the main panels of (**A**) and the specific initial conditions used in the inset panels of (**A**). Note that the ICA result and the map of the Bell-Sejnowski rule are similar to those of the Amari rule.

**Figure 4:**
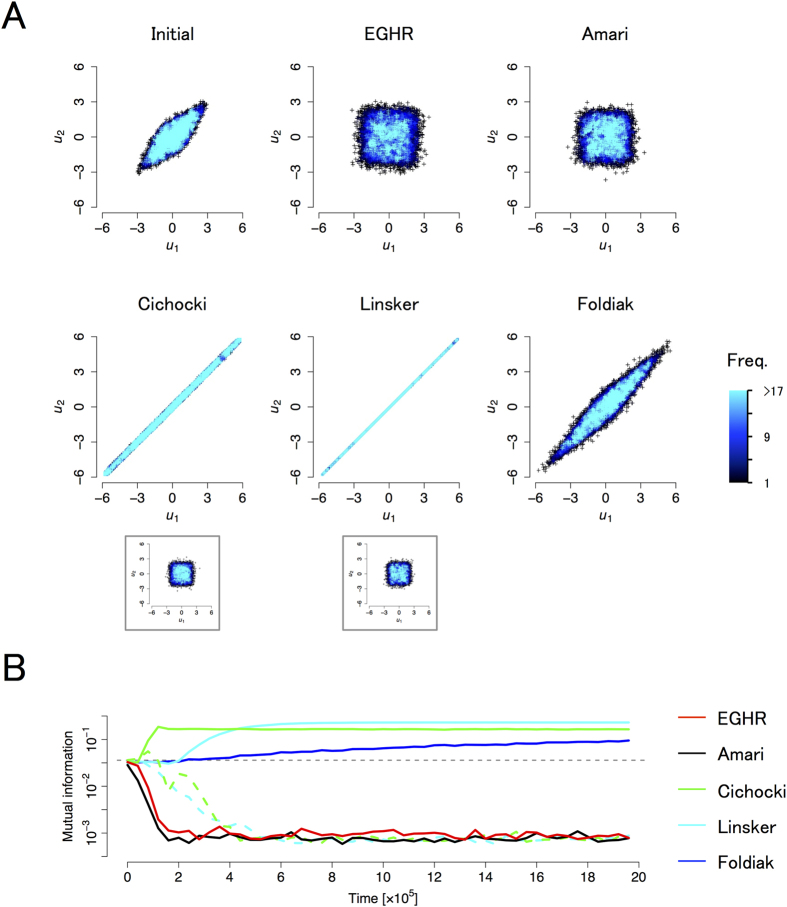
Results of ICA with a non-rotation mixing matrix and uniform source distribution. **(A)** Initial and final distributions of the outputs for each rule. Conventions are as in Fig. 3A. (B) The learning time course of each method assessed by mutual information of the outputs *I*(**u**). The EGHR and Amari rule successfully perform ICA, whereas the Linsker and Cichocki rules fail depending on the initial synaptic strength matrix, and the Foldiak rule cannot handle a non-rotation mixing matrix. Time (x-axis) is defined by *k* × ∆*t*, where *k* is the number of computing steps and ∆*t* is the time bin. Because the Linsker and Foldiak rules need a smaller time bin (∆*t* = 10 for Linsker, = 1 for Foldiak, and = 100 for others), they required more computational steps than other rules to reach a solution. The color of each learning rule is shown in the legend. Solid curves indicate the time courses of learning when started from a common initial condition *W* = (−2.2, 0; 0, −2.2). Dashed curves are the time courses for the Cichocki and Linsker rules when started from a good initial condition, that is, *W* = (−0.8, 0; 0, −0.8). A gray dashed line indicates *I*(**u**) at the beginning of learning. Other parameters are summarized in Table 1.

**Figure 5:**
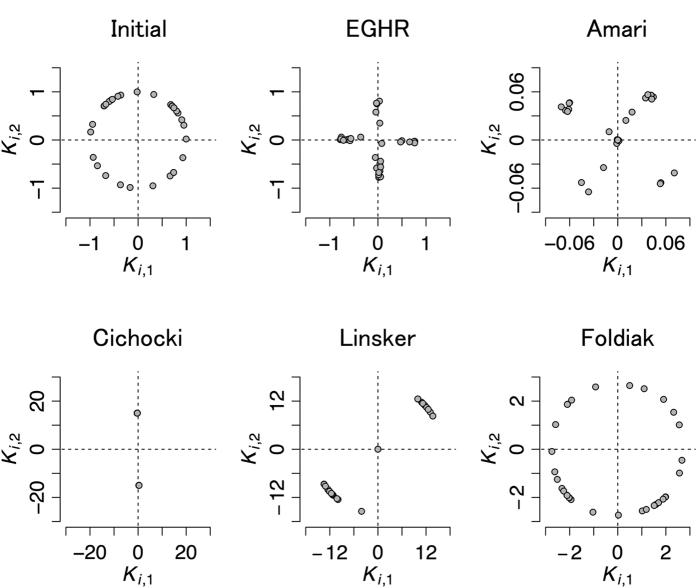
Results of ICA in the undercomplete condition. Thirty-two neurons were used to separate two sources. In each panel, the horizontal and vertical axes respectively represent the first and second elements of two-dimensional vector **k**_*i*_ (*i* = 1, … , 32), which respectively represents the responsiveness of neuron *i* to the two sources. ICA is successful if **k**_*i*_^*T*^ ∝ (1, 0) for some *i* and **k**_*i*_^*T*^ ∝ (0, 1) for others. Initially, vectors **k**_1_, …, **k**_32_ are randomly sampled on a unit circle (top left). The EGHR successfully performed ICA as indicated by **k**_1_, …, **k**_32_ directed either along the horizontal or vertical axis (top center). On the other hand, the other learning rules did not achieve ICA (other panels). See Methods for other simulation details.

All these rules are capable of performing ICA at least under restricted conditions (see Methods and S2.6). However, the stability of the ICA solutions as well as the existence of spurious solutions depends on the properties of the mixing matrix, source distribution, and how rapidly source signals change relative to the neuronal time scale. Figure 2 summarizes the performance of these learning rules, which we demonstrate in the following simulations and analyses. A tick mark indicates that a learning rule always finds an ICA solution under the corresponding condition according to our analytical (see Methods and Supplementary Note S2) and numerical (see Figs 3, 4, 5 and S1–3) analyses. Figure 2 clearly indicates that the EGHR exhibits the most reliable performance over a range of conditions.

First, we analytically investigate the linear stability of ICA solutions (Fig. 2). Because the EGHR is a gradient descent rule of a cost function *L*, a fixed point of the EGHR is stable if its second derivative *d*^2^*L* is non-negative in all parameter directions. We found that the EGHR has stable ICA solutions for a wide range of source distributions, including Laplace and uniform distributions, regardless of the form of mixing matrix *A* (see Methods and S2.2). This property is similar to the Amari rule^32^ as well as the Bell-Sejnowski rule because the two share the same cost function (see also S2.7.1). While the Linsker rule approximates the Bell-Sejinowski rule if the input changes more slowly than the neural time constant, its ICA solutions disappear if the input changes too fast (see S2.6.2). Moreover, under the Cichocki rule, some of the ICA solutions are unstable if *A* has at least one negative eigenvalue, and all ICA solutions are unstable if *A* is negative definite (see S2.7.2). Additionally, no ICA solutions exist under the Foldiak rule if *A* is a non-rotation matrix (see S2.6.3). Even if *A* is a rotation matrix, their stability depends on the detailed relationship between the source distribution and the shape of *f*_*F*_(•) (see the next section and S2.7.3).

### Numerical simulations with a rotation mixing matrix and Laplace source distribution

Here, we numerically investigate the performance of each rule. For visualization purposes, we consider again two neurons to separate two independent sources. We set the mixing matrix to rotation matrix *A* = (cos *θ*, −sin *θ*; sin *θ*, cos *θ*) with *θ* = *π*/6. The sources are generated from a Laplace distribution using the potential function *U*_*L*_ as described above. Other parameter values are summarized in Table 1.

Figure 3A depicts the initial (top left) and final (other panels) distributions of the output (*u*_1_, *u*_2_). The two output variables should become independent if each rule is successful. The EGHR and the two non-local learning rules, the Bell-Sejnowski and Amari rules, can successfully separate independent sources. Because the Bell-Sejnowski and Amari rules perform similarly, we only plotted the result of the Amari rule. In contrast, the outcome of other local learning rules, i.e., the Linsker, Cichocki, and Foldiak rules, depends on the initial condition of the synaptic strength matrix (an unsuccessful case is shown in the main panels and a successful case is shown in the inset panels).

To better understand the results, we next explore a velocity map that characterizes the dynamics of the synaptic weight matrix (Fig. 3B). Because *A* is a rotation matrix in this simulation, the synaptic weight matrix *W* = (*W*_11_, *W*_12_; −*W*_12_, *W*_11_) can be characterized by only two parameters, *W*_11_ and *W*_12_. This is because *W* remains a rotation matrix during the entire learning phase, as long as it is initially set so. On each velocity map (see Methods for computational procedures), the directions of changes in the synaptic weight matrix are indicated by color for different values of *W*_11_ and *W*_12_. Under the EGHR and Amari rule, all ICA solutions are stable and no spurious solutions exist (there are four ICA solutions at (*W*_11_, *W*_12_) = (cos *θ*, sin *θ*) for *θ* = *π*/6, 2*π*/3, 7*π*/6, and 5*π*/3). In contrast, the three conventional local rules, the Linsker, Cichocki, and Foldiak rules, have basins of attraction for spurious solutions. The Linsker rule can approximate the Bell-Sejnowki rule if the time scales of dynamical variables are set appropriately and the time bin ∆*t* is set small enough. Otherwise, it has spurious stable solutions at *W* = 0 and at infinity. This means that *W* converges to zero (or infinity) if an initial *W* is started too small (or too big). The basins of attraction for spurious solutions expand as the neuronal time constant (τ_*v*_) becomes slow relative to the sources (τ_*s*_) (see Fig. 3B for τ_*v*_/τ_*s*_ = 0.2). They eventually remove all ICA solutions if τ_*v*_ ≫ τ_*s*_ (see S2.5.1 and S2.6.2 for analyses). The Foldiak rule also has a similar spurious stable solution at *W* = 0 even if τ_*v*_ is small, and has four additional spurious solutions near the diagonal lines of the plot, indicating that it fails if *W* is initially small or at near diagonal lines. The Cichocki rule can also fail depending on the initial conditions because one of the eigenvalues of *A* is negative in this example. In the current case, two of the ICA solutions, (*W*_11_, *W*_12_) = (cos *θ*, sin *θ*) for *θ* = 2*π*/3 or 7*π*/6 are unstable, causing the synaptic strength matrix to diverge for a range of initial *W*.

### Numerical simulations with a non-rotation mixing matrix and uniform source distribution

We next numerically explore another example using a non-rotation mixing matrix *A* = (1, 0.5; 0.5; 1). We consider again two neurons to separate two independent sources for visualization purposes. Sources are generated from a uniform distribution, using the potential function *U*_*U*_ as explained above. Other parameter values are summarized in Table 1.

Figure 4A depicts the initial (top left) and final (other panels) distributions of the output (*u*_1_, *u*_2_). The two output variables should become independent if each rule is successful. Similar to the previous example, the EGHR and Amari rule successfully separate the independent sources, while the other local learning rules fail depending on the initial conditions. The Linsker rule does not work for the same reason as in the previous example. The Cichocki rule can fail regardless of the source distribution if the mixing matrix has a negative eigenvalue. The Foldiak rule generally cannot perform ICA if the mixing matrix is non-rotational (see S2.6.3).

Unlike the case with a rotation mixing matrix, we cannot easily visualize a velocity map with a non-rotation mixing matrix because *W* is characterized by more than two parameters. Instead, we monitor the time course of learning using the mutual information of outputs, defined by *I*(**u**) = ∫d**u**Prob(**u**)log[Prob(**u**)/∏_*i*_Prob(*u*_*i*_)]^12^ (Fig. 4B). This mutual information initially takes a finite value and then may converge to zero if all sources are successfully separated. Consistent with the results of Fig. 4A, the mutual information for the Linsker and Cichocki rules does not decrease if the initial synaptic strength matrix is not set appropriately. Even in the successful cases, the number of computational steps required for the Linsker and Foldiak rules is more than 10 times greater than that required for the EGHR because they need to update variable **v** in high time resolution before sources significantly change. In contrast, other learning rules, including the EGHR, require only sparse sampling of the input and yet can reach a solution within a similar physical time. (The bin size ∆*t* is set to 10 for the Linsker rule, 1 for the Foldiak rule, and 100 for other rules in Fig. 4A,B.) Thus, the EGHR’s tolerance to sparse sampling of input and the lack of a need to update extra variables (i.e., *Q* and **h)** should be highly beneficial for hardware implementation–a slow clock time for a digital device or slow dynamics for an analog device with respect to a signal of interest would be sufficient for ICA.

In sum, while all conventional local learning rules have problems even with simple examples, the EGHR can reliably perform ICA similarly to the powerful non-local learning rules. Indeed, extensive numerical simulations demonstrate that the EGHR always converges to an ICA solution for a wide range of source dimensions and randomly sampled mixing matrices (Figs S1–3). Taken together, the simplicity and robust performance of the EGHR are highly advantageous for parallel and biological computation of ICA.

### Undercomplete condition

In visual information processing, the number of neurons is usually much larger than that of the relevant sources; this case is called the undercomplete condition^16^. More generally, because a number of sources is unknown a priori and can change dynamically, it may be a good strategy to prepare a sufficient number of neurons in case they are required. Thus, if the brain performs ICA, the learning rule should be robust to the undercomplete condition. Here, we investigate whether the EGHR and conventional ICA rules can handle this condition.

We consider 32 neurons to separate two-dimensional sources. A mixing matrix *A* (32 × 2) is defined as a stack of 2 × 2 rotation matrices (see Methods for detail). To visualize the learning outcome, we define two-dimensional column vectors **k**_1_, …, **k**_32_ according to *K* = (**k**_1_, …, **k**_32_)^*T*^ = *WA*, where these vectors characterize the relation between the outputs of individual neurons and the two sources by *u*_*i*_ = **k**_*i*_^*T*^**s**. In order for the model to perform ICA, a subset of neurons must encode the first source with **k**_*i*_^*T*^ ∝ (1, 0) and another subset must encode the second source with **k**_*i*_^*T*^ ∝ (0, 1).

For all rules, the **k**_*i*_ are initially distributed randomly on a unit circle as we defined (Fig. 5 top left). We found that only the EGHR successfully found an optimal representation of sources, where **k**_1_, …, **k**_32_ were either along the horizontal or vertical axis (Fig. 5 top center). This result indicates that every neuron became specialized to one of two sources. In contrast, other learning rules were not successful in the undercomplete setting. Even with the non-local Amari rule, all the **k**_*i*_ kept mixing the two sources, thus failing to achieve ICA (Fig. 5 top right).

Indeed, we can mathematically show that the EGHR has a stable solution characterized by **k**_*i*_^*T*^ ∝ (1, 0) for some neurons and **k**_*i*_^*T*^ ∝ (0, 1) for others in a general undercomplete case (see Methods). The other rules cannot generically find such representation (see S2.8). Furthermore, the EGHR successfully separates sources even if the number of sources (more than two) dynamically changes (Supplementary Movie 1). Taken together, only the EGHR can reliably separate and extract all independent sources in the undercomplete condition.

### Application to separate natural images

**Figure 6:**
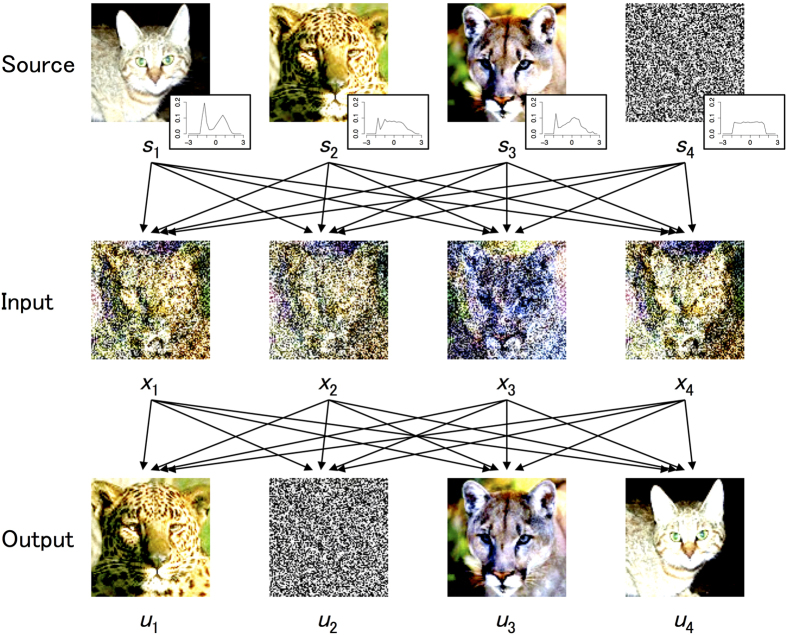
BSS of natural images. Top: Four original images as hidden signal sources. Middle: Four superposed images provided as input to the model. Bottom: Final states of the outputs of the neural network reconstructed original images. We retrieved these pictures from the Caltech101 dataset^50^ (http://www.vision.caltech.edu/Image_Datasets/Caltech101/) and processed them accordingly.

Finally, we conducted computer simulations to demonstrate a promising application of the EGHR for BSS based on natural scenes. Figure 6 displays the result of BSS using the EGHR. Three pictures of a distinct felidae animal and one white noise image were used as sources (Fig. 6 top). The color intensities of the individual pixels were processed to gray scale pixels and then converted to real numbers following^21^ (see also Methods). The four images were randomly superposed to produce four mixed images using a 4 × 4 mixing matrix (Fig. 6 middle). These four mixed images were simultaneously sampled one pixel at a time (from an identical position) and fed into four model neurons as input. The model then learned synaptic strength matrix *W* according to the EGHR. Although grayscale images are used during training, the final results are obtained by providing color images as input to the learned network.

One issue is that, while the rule needs to assume a specific distribution of sources to compute the updates of the synaptic strengths in Equation 1, this distribution is unknown in practice. Based on our observation that the EGHR is robust to the detailed shape of a source distribution (Supplementary Fig. S3), we tested Laplace and uniform distributions for *p*_0_ because only the difference of super- vs. sub-Gaussian is important. We found that the uniform distribution worked better with these images. Indeed, a posthoc analysis of the original source images confirmed that the true sources tended to obey a sub-Gaussian distribution with negative kurtosis (see inset panels in Fig. 6 top). Specifically, we ran the neural network for 2 × 10^7^ steps using a uniform distribution for *p*_0_ with learning time constant τ_*W*_ = 2 × 10^4^, and found that a series of output **u** calculated after learning successfully achieved BSS by reconstructing natural images close to the originals (Fig. 6 bottom).

**Figure 7:**
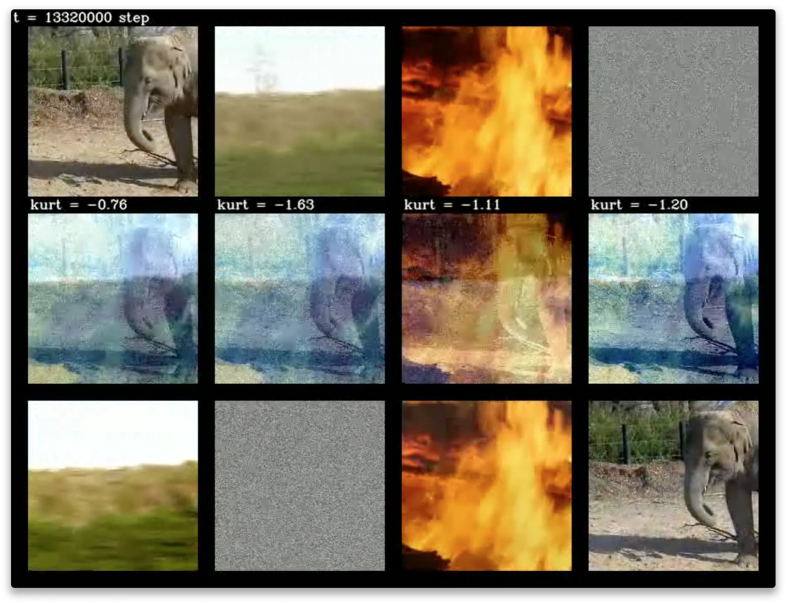
Snapshots of BSS results using movies. Top: Four original images as hidden signal sources. Middle: Four superposed images provided as input to the model. Bottom: The final states of the outputs of the neural network reconstructed the original movies well (Supplementary Movie 2). We retrieved these movies from MotionElements (https://www.motionelements.com) and processed them accordingly.

To further show the wide applicability of the EGHR, we next applied the learning rule to movies. This application was straightforward and the outcome was successful (Fig. 7; Supplementary Movie 2). This suggests the EGHR’s potential for a wide range of applications. One minor difference in this example compared to the previous one is that, while the distribution of sources remained mostly sub-Gaussian, it sporadically turned super-Gaussian. Because of this transition, a small fraction of elements of the synaptic strength matrix did not converge and kept fluctuating. Nonetheless, BSS was overall successful using *g*(**u**) and *E*(**u**) functions designed based on a uniform source distribution.

## Discussion

In this work, we proposed a new ICA rule, the EGHR, that requires only local information at each synapse for learning. We also showed that, in comparison to other ICA rules, the EGHR is the only local ICA rule that reliably works with various source statistics, mixing matrices, and number of sources.

Although we have focused on extracting independent sources in the external world in this paper, the method has a more general benefit–to adaptively organize the output of neurons nearly independent, regardless of the nature of the input. Generally, the “curse of dimensionality” makes it difficult to find a way to extract relevant information from multiple neurons^33^ unless they use a particularly simple representation^1^. The situation is known to be much easier if each neuron codes information independently^34^. Thus, the EGHR could be a general computational principle in the brain to avoid the curse of dimensionality for decoding by self-organizing nearby neurons to acquire a nearly independent information coding scheme.

In this study, we compared the EGHR with previously proposed learning rules, including local^19^,^20^,^21^ and non-local^13^,^14^,^15^ rules. We found that the EGHR is one of the most reliable learning rules among the previously proposed ICA rules in terms of the stability of genuine solutions and the absence of spurious solutions. In particular, all but the EGHR failed to extract independent sources under the undercomplete setting. Notably, the resulting stimulus representation by the EGHR utilizing all neurons, as opposed to a minimal number of neurons, is optimal according to the “infomax” principle for accurately representing sources in the presence of noise^16^,^35^. Although a non-local algorithm has been proposed to achieve ICA under the undercomplete setting^36^, to our knowledge, the EGHR is unique in achieving such an undercomplete representation by only using local computations. In the real world, the number of independent sources is unknown and may differ from one condition to the next. Hence, it is natural to prepare enough neurons in case they are needed. In this view, the EGHR’s capacity to handle undercomplete conditions is extremely beneficial in biological settings as well as in engineering applications.

Moreover, the EGHR automatically can whiten the inputs and rotate pre-whitened inputs to extract independent outputs (see S2.3–4 for additional analyses). In contrast, kurtosis-based methods such as Fast ICA^17^ assume that inputs are already whitened. This is not ideal for parallel and biological implementation of ICA because signals are typically correlated in biological systems, e.g., in the cocktail party effect. Especially, inputs are inevitably correlated in undercomplete condition. Therefore, it is a big advantage of the EGHR to be able to perform decorrelation (i.e., whitening) and ICA (i.e., increasing non-Gaussianity) simultaneously.

To date, all ICA algorithms based on neuron-like units require extensive information sharing among output neurons. However, the communication is much simpler for the EGHR than it is for other rules. While specific communication for each pair of neurons is required for other ICA algorithms, a single global signal is sufficient for the EGHR. Moreover, the frequency of communication required is also advantageous for the EGHR over the Linsker and Foldiak rules. While the Linsker and Foldiak rules require virtually continuous updating of the neural activity during learning for stability, the EGHR requires only one forward and backward information passing before stimulus significantly changes with its time constant τ_*s*_. Hence, the EGHR can successfully separate rapidly changing sources while requiring only minimal communication and processing by neurons.

In the brain, the strength of each synapse changes according to the co-activation of the pre- and post-synaptic factors, as described by Hebbian plasticity^22^,^37^ or by its variants such as spike-timing dependent plasticity (STDP)^38^,^39^. It is critical for the EGHR that the outcome of Hebbian plasticity is modulated by a global signal. Consistent with our proposal, recent experimental studies have reported the essential role of a third-factor such as GABA^23^,^24^, neuromodulators^25^,^26^,^27^,^28^,^29^, or glial signaling^30^ in directly modulating Hebbian plasticity. While the importance of three-factor learning has been shown in many computational models^40^,^41^,^42^,^43^,^44^, this is to our knowledge the first demonstration that it can play an essential role in ICA. A simple summation of surprise signal from output neurons is sufficient to compute the global error signal used for the EGHR. The surprise signal in each neuron is large if neural activity takes an unexpectedly high (or low) value, which happens more often in an unfamiliar rather than familiar environment. In this sense, the property of the surprise signal has noticeable commonality with the sensory saliency signal^45^,^46^.

**Figure 8:**
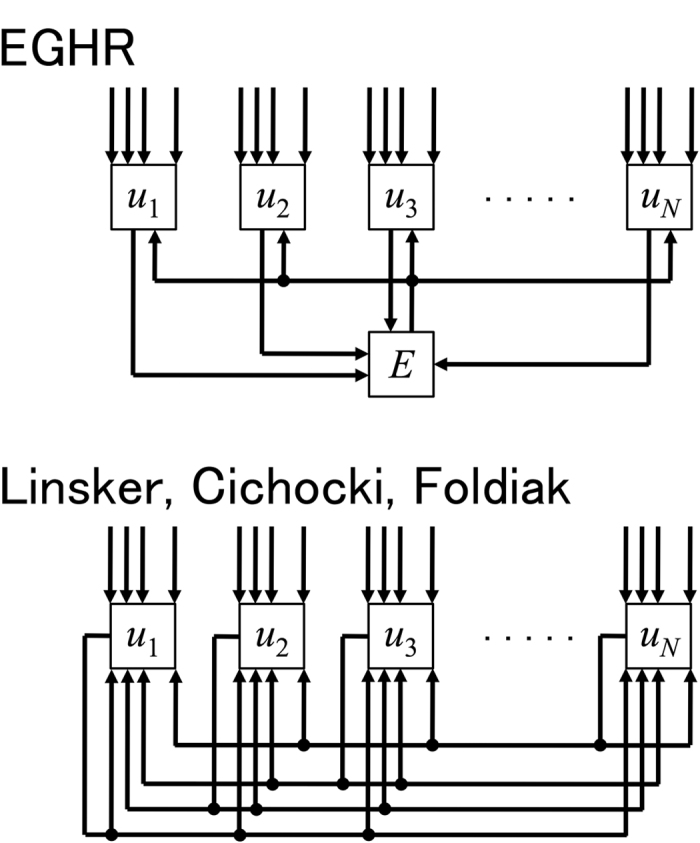
Diagrams of the proposed scheme and conventional scheme. Top: Diagram of a circuit implementing the EGHR. The EGHR requires recurrent connections, but they are only between the output neurons and the unique global factor *E*; therefore, the total number of recurrent connections is only 2*N*. Bottom: Diagram of a conventional circuit implementing local ICA rules. Conventional local ICA rules use *N* × *N* recurrent connections between all output neurons (i.e., the Linsker, Cichocki, and Foldiak rules) and their strengths need to be learned (for the Linsker and Foldiak rules). Thus, the small number of fixed recurrent connections is a significant advantage of the EGHR with respect to possible applications in neuromorphic engineering.

In addition, a simple circuit architecture of the EGHR provides an ease for neuromorphic computation. Conventional local ICA rules, including a recently proposed one^47^, use lateral inhibition to decorrelate output neurons. This requires *N* × *N* mutual connections among the output neurons, and they need to be tuned in an activity-dependent manner (Fig. 8 bottom). Biologically, recurrent connections are rather sparse (~10% or less connectivity^48^), and this limitation can reduce the performance of conventional learning rules. Exactly the same set of problems arises if conventional learning rules are implemented in neuromorphic chips. The necessity of dense, specific, and dynamic recurrent connections can easily complicate the circuit architecture. In contrast, the EGHR robustly and efficiently performs ICA, only needing the interaction between output neurons and a global factor–thus, only 2*N* non-plastic recurrent connections suffice (Fig. 8 top). This feature makes the EGHR an excellent candidate for implementation using neuromorphic technology^49^.

In summary, we developed a new local ICA rule based on a global error signal. The proposed rule performs an extremely robust ICA computation using only locally available information and a minimum number of operations. The broad applicability and easy implementation of the present rule could further advance neuromorphic computation and may reveal the principle underlying BSS computation in the brain.

## Methods

### Derivation of the proposed learning rule

The generative model of input signals is represented as **x** = *A***s**, where **s** = (*s*_1_, …, *s*_*M*_)^*T*^ and **x** = (*x*_1_, …, *x*_*N*_)^*T*^ are *M*- and *N*-dimensional column vectors of independent sources and merged inputs, respectively, and *A* is an *N* × *M* transform matrix from sources to inputs (see Fig. 1A). The true probability distribution of *s*_1_, …, *s*_*M*_ is represented as *p*(*s*_*i*_), i.e., we assume *s*_1_, …, *s*_*M*_ independently obey an identical distribution. However, the true distribution is usually unknown to the observer, so we set the prior distribution of *s*_*i*_ as *p*_0_(*s*_*i*_). The prior of **s** is represented as *p*_0_(**s**) = ∏_*i*_ *p*_0_(*s*_*i*_). In addition, the distribution of **x** is represented as *p*(**x**). The neural network model with linear firing rate is defined as **u** = *W***x**, where **u** = (*u*_1_, …, *u*_*N*_)^*T*^ is an *N*-dimensional column vector of outputs, and *W* is an *N* × *N* transform matrix from the inputs to outputs. We define the probability distribution of **u** as *q*(**u**), which is a posterior that the neural network recognizes. Unless specifically mentioned, we assume *M* = *N*. To perform infomax learning, as in the Bell-Sejnowski and Amari rules, *q*(**u**) should become the same shape as *p*_0_(**u**) because *u*_1_, …, *u*_*N*_ become independent of each other if and only if *q*(**u**) = *p*_0_(**u**)^16^. Hence, the Bell-Sejnowki and Amari rules minimize the Kullback-Leibler divergence^12^ between *q*(**u**) and *p*_0_(**u**), computed by *L*_*A*_ = *D*_*KL*_[*q*(**u**)||*p*_0_(**u**)], to evaluate the distance of two distributions^13^,^14^,^15^. The idea of the proposed method is to use another cost function that is more tractable for the neural network. The proposed method (EGHR) is derived from a cost function *L*, which is a functional of a prediction error *E*(**u**) (also known as a prior energy function). First, we define *E*(**u**) by


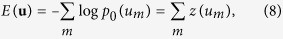


where *z*(*u*_*m*_) = −log *p*_0_(*u*_*m*_) for all *m* = 1, …, *N*. Next, we defined cost function *L* by


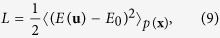


where *E*_0_ is a positive constant value defined depending on the shape of *p*_0_(**s**). The bracket 〈•〉_*p*(**x**)_ represents an expectation over input distribution *p*(**x**), that is, 〈•〉_*p*(**x**)_ = ∫ • *p*(**x**) *d***x**. For simplification, we also write 〈•〉_*p*(**x**)_ as 〈•〉. Learning in the EGHR occurs with the change in *W* and its goal is to minimize *L*, so that the dynamics of *W* are defined as the first order derivative of *L*, which is calculated as


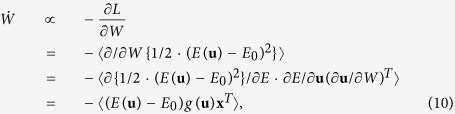


where *g*(*u*) = −dlog *p*_0_(*u*)/d*u*. Accordingly, Equation 1 is derived from Equation 9, although the learning time constant τ_*W*_ needs to be defined separately.

Equation 1 indicates that *W* is proportional to the expectation of the multiplication of global factor (*E*_0_ − *E*(**u**)) (a scalar) by the Hebbian term *g*(**u**)**x**^*T*^ (a matrix). The former can be regarded as a learning efficacy depending on **u** that is common for all neurons. Because we do not assume that *g*(*s*_*i*_) is a monotone increasing function of *s*_*i*_, the EGHR potentially can be applied to sources with multimodal distributions if the distribution is within the linear-stability condition (see the following sections). Specifically, when a source distribution is *p*_0_(*s*_*i*_) = 1/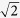
·exp(−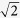 |*s*_*i*_|) (normal Laplace distribution), *g*(*s*_*i*_) becomes *g*_*L*_(*s*_*i*_) = 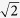 sgn(*s*_*i*_) and is approximated as 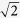 tanh(*γ s*_*i*_) for numerical calculations, where *γ* is a large positive constant. Similarly, when a source distribution is *p*_0_(*s*_*i*_) = 1/2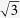 for |*s*_*i*_| < 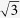 or 0 for otherwise (normal uniform distribution), *g*(*s*_*i*_) is approximated as *g*_*U*_(*s*_*i*_) = −*γ* tanh(−*γ* (*s*_*i*_ + 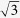)) + *γ* tanh(*γ* (*s*_*i*_ − 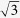)) using large positive constant *γ*.

Although *E*(**u**) in the EGHR is tractable, *H*[*q*(**u**)] for infomax rules is more difficult to calculate for both biological neurons and computers because handling of non-Gaussian distribution *q*(**u**) is required. This leads to the known difficulty of calculating the partial differential of *H*[*q*(**u**)] by *W*, i.e., ∂*H*[*q*(**u**)]/∂*W* = *W*^−*T*^, in the Bell-Sejnowski equation (see Equation 2)^13^,^14^. The EGHR instead calculates *E*(**u**)^2^, so that its partial differential 2*E*(**u**)*g*(**u**)**x**^*T*^ is more tractable than *W*^−*T*^ for neurons.

### Equilibrium point of the EGHR

We show *W* = *A*^−1^ is an equilibrium point of Equation 1. Again, we write *z*(*u*_*i*_) = −log *p*_0_(*u*_*i*_) and *E*(**u**) = ∑_*m*_ *z*(*u*_*m*_). When *W* = *A*^−1^, Equation 1 becomes *W* ∝ 〈(*E*_0_ − ∑_*m*_ *z*(*s*_*m*_))*g*(**s**)**s**^*T*^〉*A*^*T*^ since the relationship of **u** = *A*^−1^ *A***s** = **s** holds. For simplification, we assume that 〈**s**〉 = **0**. In this case, 〈*g*(**s**)〉 = −∫d**s** d*p*_0_(**s**)/d**s** = **0**. As *s*_1_, …, *s*_*N*_ independently obey an identical distribution, we obtain 〈*g*(*s*_*i*_)*s*_*j*_〉 = 〈*g*(*s*_*i*_)〉〈*s*_*j*_〉 = 0 for *i* ≠ *j*. In addition, we obtain 〈∑_*m*_ *z*(*s*_*m*_)*g*(*s*_*i*_)*s*_*j*_〉 = 0 for all *m* when *i* ≠ *j*. On the other hand, when *i* = *j*, 〈*g*(*s*_*i*_)*s*_*i*_〉 and 〈*z*(*s*_*m*_)*g*(*s*_*i*_)*s*_*i*_〉 have non-zero values. Using the relationships of 〈*g*(*s*_*i*_)*s*_*i*_〉 = 1, 〈*z*(*s*_*m*_)*g*(*s*_*i*_)*s*_*i*_〉 = 〈*z*(*s*_*m*_)〉 for *m* ≠ *i*, and 〈*z*(*s*_*i*_)*g*(*s*_*i*_)*s*_*i*_〉 = 〈*z*(*s*_*i*_)〉 + 1 (see S2.1.1), we find that 〈*g*(*s*_*i*_) *s*_*j*_〉 = δ_*ij*_ and 〈∑_*m*_ *z*(*s*_*m*_)*g*(*s*_*i*_)*s*_*j*_〉 = (*N* 〈*z*(*s*_*i*_)〉 + 1)δ_*ij*_, where δ_*ij*_ is Kronecker’s delta such that δ_*ij*_ = 1 for *i* = *j* and δ_*ij*_ = 0 for *i* ≠ *j*. Let us derive the condition such that 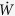 = 0 holds when *W* = *A*^−1^. Using these relationships, Equation 1 is further calculated as 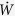
= (*E*_0_ − *N*〈*z*(*s*_*i*_)〉 − 1)*A*^*T*^ = 0. Therefore, if and only if


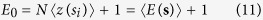


holds, *W* = *A*^−1^ is an equilibrium point of Equation 1. In this case, *E*_0_ is a constant that only depends on the shape of *p*_0_(*s*_*i*_) and the dimensions of **u**. Notably, *W* = 0 is another equilibrium point of Equation 1 if we assume *g*(**0**) = **0**. However, this turns out to be an unstable equilibrium point (see S2.2).

### With an unknown source distribution

In practical cases, however, the shape of the true distribution *p*(*s*_*i*_) is usually unknown. This means that the optimal choices for *E*_0_ and *g*, i.e., *E*_0_ = −*N*〈log *p*(*s*_*i*_)〉 + 1 and *g*(*u*) = −dlog *p*(*u*)/d*u*, are also unknown. Here, we show that the EGHR finds ICA solutions even if we choose *E*_0_ to be an arbitrary positive scalar. (While we assume the optimal *g* in this section, we show in Fig. S3 that the performance of the EGHR is also robust to the choice of *g*.) Let us consider the situation where *W* is proportional to *A*^−1^, that is, *W* = *cA*^−1^, where *c* is a positive scalar. We assume that *E*(*c***s**) is an even function of **s** and *s*_1_, …, *s*_*N*_ obey independently an identical distribution *p*(*s*_*i*_). In this condition, when *W* = *cA*^−1^, Equation 1 becomes


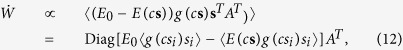


where Diag[*x*_*i*_] is a diagonal matrix in which the (*i*, *i*) elements are *x*_*i*_ and the non-diagonal elements are zero. Thus, if and only if the relationship of


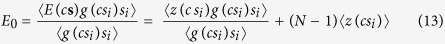


holds, *W* = *cA*^−1^ becomes an equilibrium state of Equation 1. The existence of *c* that satisfies Equation 13 is guaranteed, if we assume that *g*(**0**) = **0**, *z*(*cs*_*i*_) is a convex function, and *g*(*cs*_*i*_) is a monotonically increasing function. In this case, the right hand side of Equation 13 is a monotonically increasing function of *c* that takes 0 at *c* = 0 and tends to be ∞ as *c* approaches ∞. Therefore, for any *E*_0_ > 0, there is a positive *c* that gives the equilibrium point of the EGHR. For example, if we assume that sources obey *p*_0_(*s*_*i*_) ∝ exp(−*β* |*s*_*i*_|^*α*^) (*α* > 0, *β* > 0), then *z*(*cs*_*i*_) and *g*(*cs*_*i*_) are written as *z*(*cs*_*i*_) = *β*|*cs*_*i*_|^*α*^ = *c*^*α*^*z*(*s*_*i*_) and *g*(*cs*_*i*_) = *c*^*α*^*g*(*s*_*i*_). Therefore, *W* = *cA*^−1^ is a equilibrium point of the EGHR if and only if *E*_0_ = *c*^*α*^ (*N*〈*z*(*s*_*i*_)〉 + 1) in this example.

### Linear stability

We investigated the necessary and sufficient conditions for linear stability. In this and the following sections, we assume that the prior *p*_0_(*s*_*i*_) is the same as the true distribution of the source, *W* = *A*^−1^ is a solution of the EGHR according to Equation 11, and that *p*_0_(*s*_*i*_) is an even function of *s*_*i*_. Let us set *ρ* = cov(*z*(*s*_*i*_), *g′*(*s*_*i*_)*s*_*i*_^2^) and *ω* = cov(*z*(*s*_*i*_), *g′*(*s*_*i*_))〈*s*_*i*_^2^〉 + cov(*z*(*s*_*i*_), *s*_*i*_^2^)〈*g′*(*s*_*i*_)〉, where cov(*x*, *y*) indicates the covariance between *x* and *y*. We calculate *d*^2^*L,* the second order differential form of *L*, at *W* = *A*^−1^ as


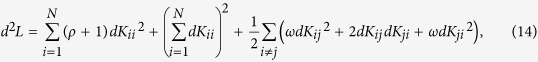


Notably, *K*_*ij*_ is an element of matrix *K* = *WA* and *dK*_*ij*_ is its differential form. We confirm that *d*^2^*L* at *W* = *A*^−1^ is definitely non-negative if and only if *ρ* > –1 and *ω* > 1 hold because a discriminant of a quadratic equation in the third term would be negative definite. Under this condition, *W* = *A*^−1^ is a stable equilibrium point and gives the minimum value of Equation 9 (see S2.2 for details).

For example, if the sources obey *p*_0_(*s*_*i*_) ∝ exp(−*β*|*s*_*i*_|^*α*^) (*α* > 0, *β* > 0), we obtain *ρ* = *α* − 1 and *ω* = 〈*s*_*i*_^2^〉〈(*g*(*s*_*i*_))^2^〉. Therefore, *d*^2^*L* is further calculated as *d*^2^*L* = ∑_*i*_ *αdK*_*ii*_^2^ + (∑_*i*_ *dK*_*ii*_)^2^ + 1/2·∑_*i*≠*j*_ (〈*s*_*i*_^2^〉〈(*z′*(*s*_*i*_))^2^〉 *dK*_*ij*_^2^ + 2*dK*_*ij*_*dK*_*ji*_ + 〈*s*_*i*_^2^〉〈(*z′*(*s*_*i*_))^2^〉*dK*_*ji*_^2^), which is definitely non-negative as long as *α* > 0 and 〈*s*_*i*_^2^〉〈(*z′*(*s*_*i*_))^2^〉 > 1. Notably, numerical simulations suggested that 〈*s*_*i*_^2^〉〈(*z′*(*s*_*i*_))^2^〉 is no less than one when *α* > 0. The above second order differential form is the same as that of the Amari rule^32^ except for the extra (∑_*i*_ *dK*_*ii*_)^2^ term, where this positive term provides additional stability for the EGHR compared to the Amari rule.

### The absence of spurious solutions and relaxation time

We analytically and numerically evaluated the absence of spurious solutions and relaxation time of the EGHR if there are more than two sources. We analytically showed that, if the source distribution is close to Gaussian, *W* = *A*^−1^ and its permutation and sign-flips are the only stable equilibrium points of the EGHR (see S2.3 and S2.4 for details).

We then numerically confirmed that there was no local minimum found when the source obeyed either a Laplace or uniform distribution by calculating the relaxation time of *W* to a fixed point. Figs S1–3 graph the relaxation time with a variety of transform matrices (Fig. S1), source dimensions (Fig. S2), and presumed source distribution shapes (Fig. S3).

### Undercomplete condition

We investigate the dynamics in the case where the output dimension dim(**u**) = *N* is larger than that of sources dim(**s**) = *M*, that is, *N* > *M*. The input dimension dim(**x**) is the same as dim(**u**) = *N*. As a special case, let us assume that **x** and **u** are 2*M*-dimensional column vectors (*N* = 2*M*). Then *W* is a 2*M* × 2*M* square matrix, and *A* = (*A*_1_^*T*^, *A*_2_^*T*^)^*T*^ is a 2*M* × *M* block matrix. Similar to the previous section, we define a 2*M* × *M* vertically long matrix *K* = *WA*. From the infomax viewpoint, the optimal solutions comprise *K* = (*Λ*_1_, *Λ*_2_)^*T*^ and its permutations and sign-flips, where *Λ*_1_ and *Λ*_2_ are non-zero diagonal matrices. This is because the representation using two neurons per source (**u** = *K***s** = (**s**^*T*^*Λ*_1_, **s**^*T*^*Λ*_2_)^*T*^) can more accurately convey the information of the sources than using a single neuron per source (**u** = (**s**^*T*^*Λ*_1_, **0**^*T*^)^*T*^) if there is a small amount of background noise. Therefore, when *K* = (*Λ*_1_, *Λ*_2_)^*T*^, Equation 1 becomes


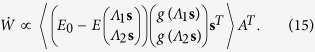


If we assume *K* = (*I*, *I*)^*T*^, since the elements of **s** are independent of each other, Equation 15 is further calculated as


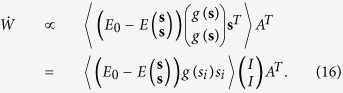


Similar to the case where dim(**s**) = dim(**u**), Equation 16 is in the equilibrium point if *E*_0_ satisfies 〈(*E*_0_ − *E*((**s**^*T*^, **s**^*T*^)^*T*^))*g*(*s*_*i*_)*s*_*i*_〉 = 0. Therefore, *K* = (*I*, *I*)^*T*^ is an ICA solution of the EGHR. The same explanation can be applied to any case where dim(**u**) > dim(**s**). Linear stability in the undercomplete condition also can be shown in a similar way.

### For Fig. 1

We used a two-dimensional colored (Fig. 1A) and white (Fig. 1B) noises obeying a Laplace distribution. A transform matrix *A* was defined as *A* = (1, 0.5; 0.5, 1) (Fig. 1A) or *A* = (cos 6/*π*, −sin 6/*π*; sin 6/*π*, cos 6/*π*) (Fig. 1B). The initial state of connection strength matrix *W* was set to *W* = (1.5, 0; 0, 1.5), i.e., the initial *u*_1_ and *u*_2_ were not independent. A learning time constant of *τ*_*W*_ = 10^3^ and a time resolution of Δ*t* = 100 were used. Simulations were conducted over *T* = 2 × 10^6^ steps.

### For Fig. 3A

A two-dimensional colored noise obeying a Laplace distribution with zero mean and a variance of one, generated by Equation 7 with *U*_*L*_(*s*) = 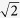
|*s*_*i*_|, was used. The mixing matrix was set to rotation matrix *A* = (cos *θ*, –sin *θ*; sin *θ*, cos *θ*) with *θ* = *π*/6. The synaptic strength matrix was initially started from *W* = (−1.5, 0; 0, −1.5) in the main panels. In the insets, final distributions with the desired initial conditions were used, namely *W* = (1.5, 0; 0, 1.5) initially for the Cichocki rule, *W* = (−0.8, 0; 0, −0.8) initially for the Linsker rule, and *W* = (1.5 cos *π*/6, −1.5 sin *π*/6; 1.5 sin *π*/6, 1.5 cos *π*/6) initially for the Foldiak rule. A common learning time constant *τ*_*W*_ = 10^3^ was used for the EGHR, Amari, and Cichocki rules. For the Linsker and Foldiak rules, *τ*_*W*_ = 10^4^ and 10^6^ were used, respectively. The time resolutions for each rule were Δ*t* = 100 for the EGHR, Amari, and Cichocki rules, Δ*t* = 10 for the Linsker rule, and Δ*t* = 1 for the Foldiak rule. Simulations continued for *T* = 2 × 10^6^ steps. For the Foldiak rule, *f*_*F*_(*u*_*i*_) = 1/(1 + exp (−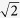
*u*_*i*_^3^))/0.225 was used. To prevent the divergence of *W*, whenever ∑_*j*_ *W*_*ij*_^2^ exceeded 4^2^, (*W*_*i*1_, …, *W*_*iN*_) was rescaled to (*W*_*i*1_, …, *W*_*iN*_)·4/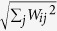. See also Table 1 for parameter details.

### For Fig. 3B

A numerical integration along a probability distribution of source *p*_0_(**s**) was used instead of the Monte Carlo sampling method to calculate the expectations. A spatial resolution of *ds* = 0.1 and a range of −20 ≤ *s*_*i*_ ≤ 20 were used for all *i*. Parameters *W*_11_, and *W*_12_ were moved within −1.5 ≤ *W*_11_ < 1.5, and −1.5 ≤ *W*_12_ < 1.5 in increments of 0.05 steps. For the Foldiak rule, *f*_*F*_(*u*_*i*_) = 1/(1 + exp(−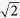
*u*_*i*_^3^))/0.225 was used. For the numerical calculation, we analytically simplified the Linsker rule as


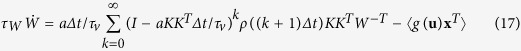


and the Foldiak rule as


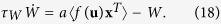


See S2.5.1 and S2.5.2 for derivation details. Note that *ρ*(*t*) is the auto-correlation of a signal train generated from Equation 7. We define Equations 17 and 18 to be the reduced Linsker (R-Linsker) and the reduced Foldiak (R-Foldiak) rules, respectively. The numerical calculation in Fig. 3B is based on this R-Lisnker and R-Foldiak rules.

### For Fig. 4A

Source signals were independently drawn from a two-dimensional colored uniform distribution with zero mean and a variance of one, generated by Equation 7 with *U*_*U*_(*s*) = 1/(2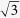) for |*s*_*i*_| ≤ 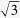 and *U*_*U*_(*s*_*i*_) = 0 for |*s*_*i*_| > 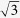. A non-rotation transform matrix *A* = (1, 0.5; 0.5, 1) was used. In the main panels, the initial and final distributions with *W* = (−2.2, 0; 0, −2.2) initially are shown. In the insets, the final distributions with desired initial conditions, namely *W* = (−0.8, 0; 0, −0.8) initially for the Cichocki and Linsker rules, were used. For the Foldiak rule, *f*_*F*_(*u*_*i*_) = 1/(1 + exp(−100*u*_*i*_)) was used. Parameters other than *U*(*s*), *p*_0_(**s**), *A*, initial *W*, and *f*_*F*_(*u*_*i*_) are the same as in Fig. 3A.

### For Fig. 4B

Mutual information between *u*_1_ and *u*_2_, *I*(**u**) = 〈log *q*(**u**) −log *q*(*u*_1_) −log *q*(*u*_2_)〉_*q*(**u**)_, was used for evaluation, where *q*(**u**), *q*(*u*_1_), and *q*(*u*_2_) were calculated using a histogram method. The parameters are the same as in Fig. 4A.

## Additional Information

**How to cite this article**: Isomura, T. and Toyoizumi, T. A Local Learning Rule for Independent Component Analysis. *Sci. Rep.* **6**, 28073; doi: 10.1038/srep28073 (2016).

## Supplementary Material

Supplementary Information

Supplementary Movie 1

Supplementary Movie 2

Supplementary Information

## Figures and Tables

**Table 1:** Model parameters.

Name	Symbol	Value
Time resolution of source	*dt*	1
Simulation time	*T*	2 × 10^6^
Time constant of source	*τ*_*s*_	50
Time resolution of algorithm	Δ*t*	100 for EGHR, Amari, Cichocki 10 for Linsker 1 for Foldiak
Time constant of *W*	*τ*_*W*_	10^3^ for EGHR, Amari, Cichocki 10^4^ for Linsker 10^6^ for Foldiak
Time constant of *Q* and h	*τ*_*Q*_	*τ*_*W*_/10 for Linsker, Foldiak
Time constant of h	*τ*_*h*_	*τ*_*W*_/10 for Linsker, Foldiak
Time constant of v	*τ*_*v*_	10 for Linsker, Foldiak
Amplification factor	*a*	1 for Linsker 1.1 for Foldiak
Mean of v	*b*	〈*f*_*F*_(*s*_*i*_)〉_*p*0(*si*)_ for Foldiak
