# Supplementary material for: A Local Learning Rule for Independent Component Analysis
Source: Sci Rep. 2016 Jun 21;6:28073. doi: 10.1038/srep28073 (PMC4914970; doi:10.1038/srep28073)
Supplement: Supplementary Information [file srep28073-s1.pdf]

Supplementary Information

A Local Learning Rule for Independent Component Analysis

Takuya Isomura and Taro Toyoizumi

S1 Supplementary Tables, Figures, Movies, and Source codes
S2 Supplementary Notes

S1 Supplementary Tables, Figures, Movies, and Source codes

Table S1. Dynamics of ICA rules.

	Dynamics of W	Dynamics of $K = W A$	Dynamics of $K = I + \varepsilon J$
EGHR	$\dot{W} \propto \langle (E_0 - E(\mathbf{u}))\, g(\mathbf{u})\, \mathbf{x}^T \rangle$	$\dot{K} \propto \langle (E_0 - E(\mathbf{u}))\, g(\mathbf{u})\, \mathbf{s}^T \rangle A^T A$	$\dot{J} \propto \langle \{(U_0 - U(\mathbf{s}))\, \Lambda$ $- 1/N\, g(\mathbf{s})\, g(\mathbf{s})^T\}\, J\, \mathbf{s}\, \mathbf{s}^T \rangle A^T A$
Bell-Sejnowski	$\dot{W} \propto \langle W^{-T} - g(\mathbf{u})\, \mathbf{x}^T \rangle$	$\dot{K} \propto \langle K^{-T} - g(\mathbf{u})\, \mathbf{s}^T \rangle A^T A$	$\dot{J} \propto -(J^T + \langle \Lambda\, J\, \mathbf{s}\, \mathbf{s}^T \rangle)\, A^T A$
Amari	$\dot{W} \propto \langle I - g(\mathbf{u})\, \mathbf{u}^T \rangle\, W$	$\dot{K} \propto \langle I - g(\mathbf{u})\, \mathbf{u}^T \rangle\, K$	$\dot{J} \propto -(J^T + \langle \Lambda\, J\, \mathbf{s}\, \mathbf{s}^T \rangle)$
Cichocki	$\dot{W} \propto \langle I - g(\mathbf{u})\, \mathbf{u}^T \rangle$	$\dot{K} \propto \langle I - g(\mathbf{u})\, \mathbf{u}^T \rangle\, A$	$\dot{J} \propto -(J^T + \langle \Lambda\, J\, \mathbf{s}\, \mathbf{s}^T \rangle)\, A$
Linsker R-Linsker	$\dot{W} \propto \langle a\, \mathbf{v}\, \mathbf{x}^T - g(\mathbf{u})\, \mathbf{x}^T \rangle$ $\dot{W} \propto \langle c_L(K)\, W^{-T} - g(\mathbf{u})\, \mathbf{x}^T \rangle$	$\dot{K} \propto \langle a\, \mathbf{v}\, \mathbf{s}^T - g(\mathbf{u})\, \mathbf{s}^T \rangle A^T A$ $\dot{K} \propto \langle c_L(K)\, K^{-T} - g(\mathbf{u})\, \mathbf{s}^T \rangle A^T A$	 $\dot{J} \propto -(c_L(K)\, J^T + \langle \Lambda\, J\, \mathbf{s}\, \mathbf{s}^T \rangle)\, A^T A$
Foldiak R-Foldiak	$\dot{W} \propto \langle a\, \mathbf{v}\, \mathbf{x}^T - \mathrm{Diag}[\mathbf{v}]\, W \rangle$ $\dot{W} \propto \langle a\, f_F(\mathbf{u})\, \mathbf{x}^T \rangle - b\, W$	$\dot{K} \propto \langle a\, \mathbf{v}\, \mathbf{s}^T A^T A - \mathrm{Diag}[\mathbf{v}]\, K \rangle$ $\dot{K} \propto \langle a\, f_F(\mathbf{u})\, \mathbf{s}^T \rangle A^T A - b\, K$	 $\dot{J} \propto \langle a\, \mathrm{Diag}[f_F'(\mathbf{s})]\, J\, \mathbf{s}\, \mathbf{s}^T \rangle - b\, J$

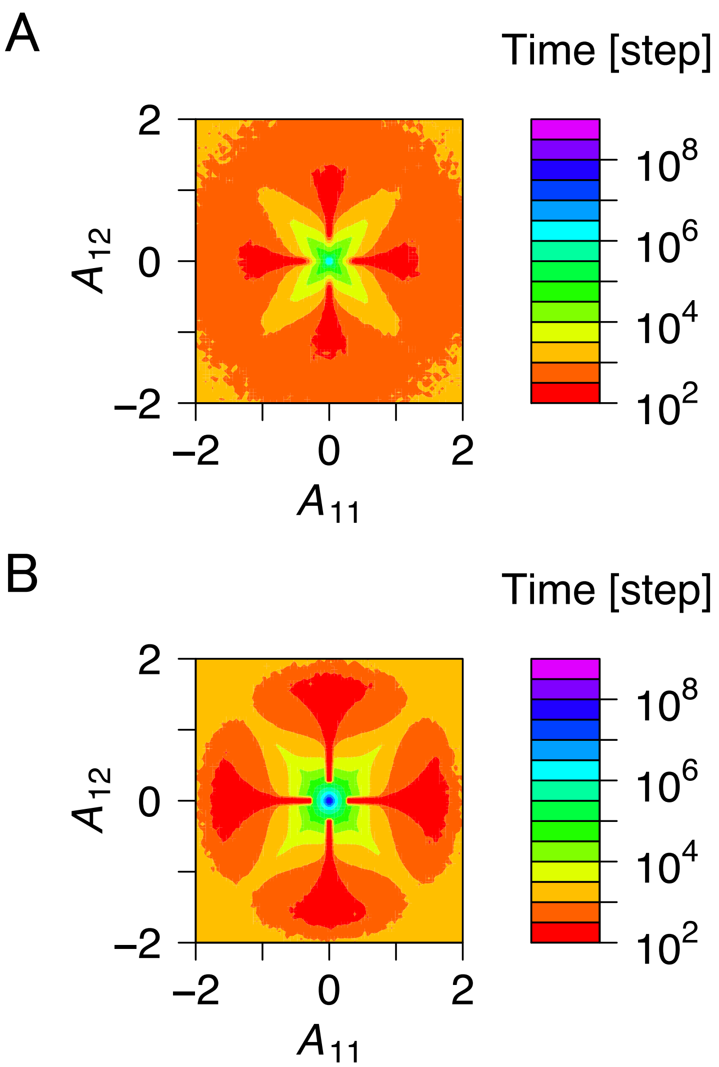

Figure S1. The absence of spurious solutions and relaxation time of the EGHR with sources conforming to Laplace (A) and uniform (B) distributions. We numerically confirmed that there was no local minimum. We considered a two-dimensional source (dim $\mathbf{s} = 2$) and transform matrix A that is defined as a rotation and scaling matrix $A = (A_{11}, A_{12}; -A_{12}, A_{11})$. We allowed W to learn according to the EGHR to ensure that the elements of $\mathbf{u} = W\mathbf{x}$ were independent of each other. The learning time constant was defined as $\tau_W = 2 \times 10^2$. We defined the relaxation time as the time needed by the rule to perform ICA. ICA ability was evaluated using the maximum value of the ratio of first to second maximum values for each row and column of matrix $K = WA$. Thus, for the ith row, we compared $|K_{ik}/K_{il}|$ with threshold $e_{th} = 0.01$, where $|K_{ik}|$ and $|K_{il}|$ are the maximum and second maximum absolute values in the ith row. We also compared

$|K_{kj}/K_{lj}|$ with threshold e_{th}, where $|K_{kj}|$ and $|K_{lj}|$ are the maximum and second maximum absolute values in the jth column We defined the relaxation time as the time at which all $|K_{ik}/K_{il}|$ and $|K_{kj}/K_{lj}|$ for all i, j first become smaller than e_{th}. This was evaluated once every 100 steps. Parameters A_{11}, and A_{12} were moved within $-2 \leq A_{11} \leq 2$, and $-2 \leq A_{12} \leq 2$ in increments of 0.05 steps. Relaxation times were calculated 100 times for each parameter set and the means are shown in graphs. The upper bound of the simulation time was defined by $T = 4 \times 10^7$. In all cases, W started to form an identical matrix and converged to one of the ICA solutions before the T step.

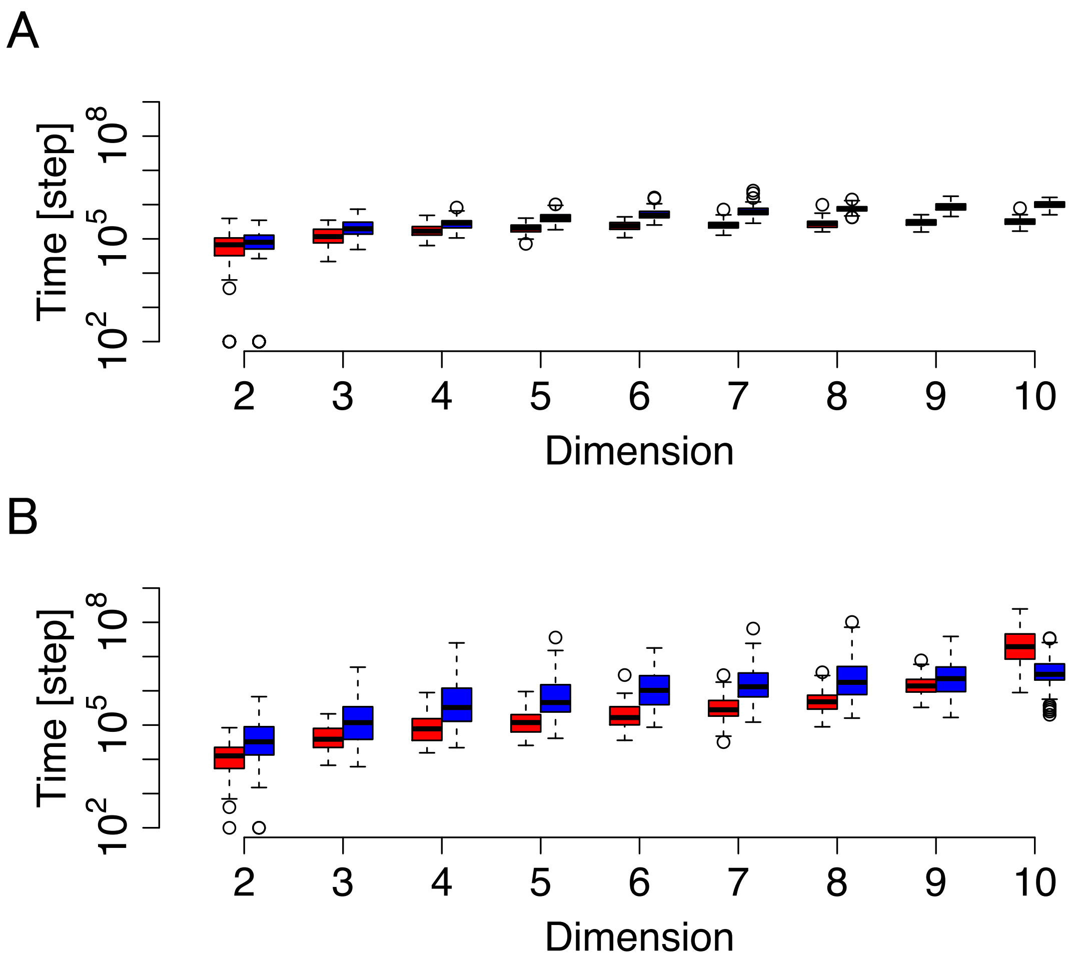

Figure S2. Dimension dependency of EGHR relaxation time. **(A)** Dimension dependency of relaxation time of Laplace (red) and uniform (blue) distributions with rotation matrix A. We assumed transform matrix A to be a rotation matrix that was rotated for all possible axes ($N(N-1)/2$ axes) with randomly selected angles θ_k from $-\pi/4 \leq \theta_k < \pi/4$, where N is the common dimension of sources, inputs, and output. Note that k is the index of one of the $N(N-1)/2$ axes. The relaxation time was calculated using the same criterion as in Fig S1. A learning time constant of $\tau_W = 2 \times 10^4$ was used. The upper bound of the simulation time was $T = 1 \times 10^9$. Red boxes represent the median of the relaxation time distributions for a Laplace distribution, while blue boxes represent those for a uniform distribution. Further, W was started from an $N \times N$-dimensional identical matrix. **(B)** Dimension dependency of the relaxation time of Laplace (red) and uniform (blue) distributions. We assumed A to be a random matrix, where each element of A was randomly selected from a normal Gaussian distribution,

$A_{ij} \sim N(0, 1)$. Note that only a matrix A whose determinant was larger than $\exp(-N/2)$ was used for the simulations. A learning time constant of $\tau_W = 2 \times 10^3$ was used. Other parameters are the same as in (A). The results reveal that although relaxation time increased with both distributions as source dimension increased, it was within a finite time and the rate of the increase was slower than an exponential increase.

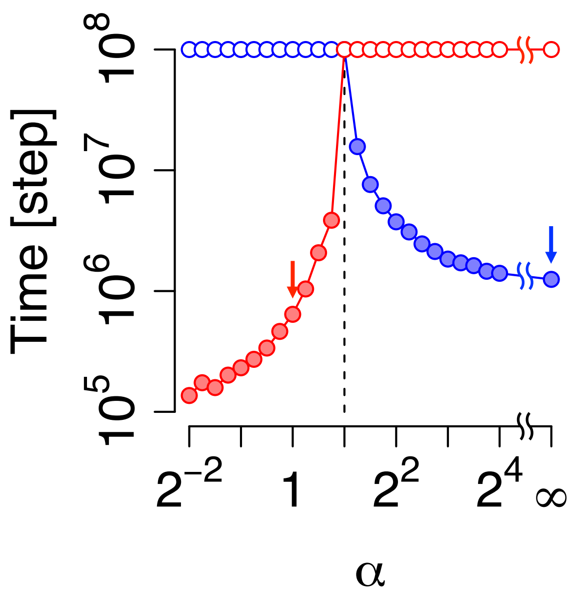

Figure S3. Robustness of the EGHR to a choice of nonlinear function g. We generate a two-dimensional source obeying $p(s_i) \propto \exp(-\beta\,|s_i|^{\alpha})$ ($\alpha > 0$, $\beta > 0$) and assume A to be a rotation matrix, $A = (\cos\pi/6, -\sin\pi/6; \sin\pi/6, \cos\pi/6)$. Note that β was defined so that the variance of s_i was one. We investigate how the choices of g, the ones designed for $\alpha = 1$ and ∞, influence the relaxation time of EGHR to one of the ICA solutions for a range of α. Relaxation time was calculated using the same criterion in Fig S1. A learning time constant of $\tau_W = 1 \times 10^5$ was used. The upper bound of simulation time was $T = 1 \times 10^8$, and W was started from an identical matrix. The red circles represent relaxation times when we used a non-linear function $g_L(u_i)$ that was optimized for a Laplace distribution (thus $\alpha = 1$; see the red arrow in the figure). Blue circles represent relaxation times when we used a non-linear function $g_U(u_i)$ that was optimized for a uniform distribution ($\alpha = \infty$; blue arrow). Filled circles indicate ICA was successful with the non-linear function before the T-th step, while open circles indicate that ICA was not achieved before the T-th step. The details of $g_L(u_i)$ and $g_U(u_i)$ are described in Methods. When the source obeys a Gaussian distribution ($\alpha = 2$; a dashed line), both $g_L(u_i)$ and $g_U(u_i)$ fail to achieve ICA, since any rotation matrix W makes the elements of $\mathbf{u}$ independent of each other when $\alpha = 2$.

Supplementary movie legends

Supplementary Movie 1. Performance of the EGHR in undercomplete condition. The EGHR successfully separates sources even if the number of sources (more than two) dynamically changes.

Supplementary Movie 2. Blind source separation results using movies. Top: Four original images as hidden signal sources. Middle: Four superposed images provided as input to the model. Bottom: The final states of the outputs of the neural network reconstructed the original movies well. We retrieved these movies from MotionElements (https://www.motionelements.com) and processed them accordingly.

Supplementary source codes

Supplementary Source Code 1. A MATLAB source code of EGHR that demonstrates 2-dimensional ICA (see the Figure 1B legend and Methods for details).

S2 Supplementary Notes

S2.1 Lemma

S2.1.1 $\langle f(s_i) g(s_i)\rangle_{p0(si)} = \langle f'(s_i)\rangle_{p0(si)}$.

[Proof]

When we assume that $f(s_i)$ is an arbitrary function of s_i and $g(s_i) := -\partial/\partial s_i \log p_0(s_i) = -p_0'(s_i)/p_0(s_i)$, we obtain

$$\begin{aligned}\langle f(s_i)\, g(s_i)\rangle_{p0(si)} &= \int f(s_i)\, g(s_i)\, p_0(s_i)\, ds_i \\ &= -\int f(s_i)\, p_0'(s_i)\, ds_i = -[f(s_i)\, p_0(s_i)] + \int f'(s_i)\, p_0(s_i)\, ds_i \\ &= \langle f'(s_i)\rangle_{p0(si)}. \qquad \text{(S1)}\end{aligned}$$

From Eq. (S1), when we suppose $z_i := -\log p_0(s_i)$ and $z_i' = \partial z_i/\partial s_i = g(s_i)$, we obtain the following equations:

$$\begin{aligned}&\langle z_i'\, s_i\rangle_{p0(si)} = \langle 1\rangle_{p0(si)} = 1, \\ &\langle z_i\, z_i'\, s_i\rangle_{p0(si)} = \langle z_i + z_i'\, s_i\rangle_{p0(si)} = \langle z_i\rangle_{p0(si)} + 1, \\ &\langle z_i'^2\, s_i^2\rangle_{p0(si)} = \langle z_i''\, s_i^2 + 2\, z_i'\, s_i\rangle_{p0(si)} = \langle z_i''\, s_i^2\rangle_{p0(si)} + 2, \\ &\langle z_i'^2\rangle_{p0(si)} = \langle z_i''\rangle_{p0(si)}. \qquad \text{(S2)}\end{aligned}$$

S2.1.2 When a function $\varphi(s_1, \ldots, s_N)$ is an odd function and $p(s_i)$ is an even function of s_i, $\langle \varphi(s_1, \ldots, s_N)\rangle_{p(\mathbf{x})} = 0$.

[Proof]

$$\begin{aligned}\langle \varphi(s_1, \ldots, s_N)\rangle_{p(\mathbf{x})} &= \langle \varphi(s_1, \ldots, s_N)\rangle_{p0(\mathbf{s})} \\ &= \int\ldots\int \varphi(s_1, \ldots, s_N)\, p_0(s_1)\ldots p_0(s_N)\, ds_1\ldots ds_N \\ &= \int\ldots\int \{\int \varphi(s_1, \ldots, s_N)\, p(s_i)\, ds_i\}\, p(s_1)\ldots p(s_N)\, ds_1\ldots ds_N \\ &= \int\ldots\int \{0\}\, p(s_1)\ldots p(s_N)\, ds_1\ldots ds_N = 0. \qquad \text{(S3)}\end{aligned}$$

S2.1.3 $\langle |s_i|^{\alpha+2}\rangle_{N(si;\,0,\,1)} = (\alpha + 1)\, \langle |s_i|^{\alpha}\rangle_{N(si;\,0,\,1)}$.

[Proof]

We define $\langle \bullet\rangle_{N(si;\,0,\,1)}$ to be the expectation over the normal Gaussian distribution $N(s_i; 0, 1)$, i.e., $\langle \bullet\rangle_{N(si;\,0,\,1)} = \int \bullet\, N(s_i; 0, 1)\, ds_i$. We obtain

$$\langle |s_i|^{\alpha+2} \rangle_{N(si;\,0,\,1)} = \int |s_i|^{\alpha+2} \cdot (2\pi)^{-1/2} \exp(-s_i^2/2)\, ds_i$$
$$= \int -|s_i|^{\alpha}\, s_i \cdot \{(2\pi)^{-1/2} \exp(-s_i^2/2)\}'\, ds_i$$
$$= -[|s_i|^{\alpha}\, s_i \cdot (2\pi)^{-1/2} \exp(-s_i^2/2)]$$
$$+ \int \{\alpha\, |s_i|^{\alpha-1} \operatorname{sgn}(s_i)\, s_i + |s_i|^{\alpha}\} \cdot (2\pi)^{-1/2} \exp(-s_i^2/2)\, ds_i$$
$$= \int (\alpha + 1)\, |s_i|^{\alpha} \cdot (2\pi)^{-1/2} \exp(-s_i^2/2)\, ds_i$$
$$= (\alpha + 1) \langle |s_i|^{\alpha} \rangle_{N(si;\,0,\,1)}. \tag{S4}$$

Similarly, from Eq. (S4),

$$\langle |s_i|^{\alpha+4} \rangle_{N(si;\,0,\,1)} = \{(\alpha + 2) + 1\} \langle |s_i|^{\alpha+2} \rangle_{N(si;\,0,\,1)}$$
$$= (\alpha + 3)\, (\alpha + 1) \langle |s_i|^{\alpha} \rangle_{N(si;\,0,\,1)}. \tag{S5}$$

S2.2 Linear stability of the EGHR

The aim of this section is to determine the necessary and sufficient condition for ICA solution $W = A^{-1}$ to become a stable equilibrium point of the EGHR. The ICA solution is stable if and only if all terms in d^2L, the second differential form of the cost function of the EGHR, are non-negative. Let us define a matrix K as $K = WA$, so that $\mathbf{u}$ is rewritten as $\mathbf{u} = K\mathbf{s}$. We suppose $z_i := -\log p_0(s_i)$, $z_i' = \partial z_i/\partial s_i = g(s_i)$, and $z_i'' = \partial^2 z_i/\partial s_i^2 = g'(s_i)$. First, we consider an analogy between the EGHR and Amari rule [15]. The cost function of the Amari rule L_A is defined by $L_A = D_{KL}[q(\mathbf{u})\| \, p_0(\mathbf{u})] = \langle \log q(\mathbf{u}) - \log p_0(\mathbf{u})\rangle = E(\mathbf{u}) - H[q(\mathbf{u})]$, and the expectations of its first and second differential forms at $W = A^{-1}$ are $dL_A = 0$ and $d^2L_A = \sum_i (1 + \langle s_i^2 z_i''\rangle)dK_{ii}^2 + 1/2\sum_{i\neq j}(\langle s_i^2\rangle\langle z_i''\rangle dK_{ij}^2 + 2dK_{ij}dK_{ji} + \langle s_i^2\rangle\langle z_i''\rangle dK_{ji}^2)$ [32]. Therefore, the necessary and sufficient conditions for the Amari equation to become linearly stable are $\langle s_i^2 z_i''\rangle > -1$ and $\langle s_i^2\rangle\langle z_i''\rangle > 1$. Similarly, dL, the first differential form of L, is calculated as

$$
\begin{aligned}
dL &= \sum_{i=1}^{N}\sum_{j=1}^{N} \frac{\partial L}{\partial K_{ij}} dK_{ij} \\
&= \langle \sum_{i=1}^{N}\sum_{j=1}^{N} \frac{1}{2}\frac{\partial}{\partial K_{ij}} (E(\mathbf{u}) - E_0)^2 \, dK_{ij}\rangle \\
&= \langle \sum_{i=1}^{N}\sum_{j=1}^{N} \frac{1}{2}\frac{\partial}{\partial E} (E(\mathbf{u}) - E_0)^2 \, \frac{\partial E}{\partial u_i} \, \frac{\partial u_i}{\partial K_{ij}} \, dK_{ij}\rangle \\
&= \langle \sum_{i=1}^{N}\sum_{j=1}^{N} (E(\mathbf{u}) - E_0)\, g(u_i)\, s_j \, dK_{ij}\rangle \\
&= \mathrm{tr}(\langle (E(\mathbf{u}) - E_0)\, g(\mathbf{u})\, \mathbf{s}^T\rangle \, dK^T).
\end{aligned} \tag{S6}
$$

Eq. (S6) is a differential form of the EGHR. As described in Methods, the expectation of Eq. (S6) at $W = A^{-1}$ (thus, $K = A^{-1}A = I$) is zero if we adequately choose E_0 (see Eq. (11)). The second order differential form of L, d^2L, is then calculated as

$$
\begin{aligned}
d^2L &= \sum_{k=1}^{N}\sum_{l=1}^{N} \frac{\partial (dL)}{\partial K_{kl}} dK_{kl} \\
&= \langle \sum_{i=1}^{N}\sum_{j=1}^{N}\sum_{k=1}^{N}\sum_{l=1}^{N} \frac{\partial}{\partial K_{kl}} (E(\mathbf{u}) - E_0)\, g(u_i)\, s_j \, dK_{ij}\, dK_{kl}\rangle \\
&= \langle \sum_{i=1}^{N}\sum_{j=1}^{N}\sum_{k=1}^{N}\sum_{l=1}^{N} \{ \frac{\partial}{\partial K_{kl}} (E(\mathbf{u}) - E_0)\, g(u_i)\, s_j + (E(\mathbf{u}) - E_0) \frac{\partial g(u_i)}{\partial K_{kl}} s_j\} \, dK_{ij}\, dK_{kl}\rangle
\end{aligned}
$$

$$= \langle \sum_{i=1}^{N}\sum_{j=1}^{N}\sum_{k=1}^{N}\sum_{l=1}^{N} \{ \frac{\partial E}{\partial u_k}\frac{\partial u_k}{\partial K_{kl}} g(u_i)\, s_j + (E(\mathbf{u}) - E_0)\frac{\partial g(u_i)}{\partial u_k}\frac{\partial u_k}{\partial K_{kl}} s_j\}\, dK_{ij}\, dK_{kl}\rangle$$

$$= \sum_{i=1}^{N}\sum_{j=1}^{N}\sum_{k=1}^{N}\sum_{l=1}^{N} \langle g(u_k)\, s_l\, g(u_i)\, s_j + (E(\mathbf{u}) - E_0)\frac{\partial g(u_i)}{\partial u_k} s_l\, s_j\rangle\, dK_{ij}\, dK_{kl}. \quad \text{(S7)}$$

If $K = 0$ and $g(\mathbf{0}) = \mathbf{0}$, we obtain $d^2L = (E(\mathbf{0}) - E_0)z_i''|_{ui=0}\langle s_i^2\rangle\sum_i\sum_j dK_{ij}^2$, which is non-positive when $(E(\mathbf{0}) - E_0)z_i''|_{ui=0} < 0$. Therefore, $K = 0$ is an unstable equilibrium point if $p_0(s_i)$ is peaked at s_i=0 and $z_i = -\log p_0(s_i)$ is convex. On the other hand, if $K = I$, then $\mathbf{u} = \mathbf{s}$ and

$$\langle g(u_i)\, g(u_k)\, s_j\, s_l + (E(\mathbf{u}) - E_0)\frac{\partial g(u_i)}{\partial u_k} s_j\, s_l\rangle$$

$$= \langle g(s_i)\, g(s_k)\, s_j\, s_l + \delta_{ik}\, (E(\mathbf{s}) - E_0)\frac{\partial g(s_i)}{\partial s_i} s_j\, s_l\rangle \quad \text{(S8)}$$

hold.

Case 1. For $i \neq k$, the second term is zero. Thus, Eq. (S8) is calculated as $\langle g(s_i)g(s_k)s_js_l\rangle$, which becomes $\langle g(s_i)s_i\rangle^2$ for ($i = j \neq k = l$) or ($i = l \neq j = k$), and becomes zero otherwise since $\langle s_i\rangle = \langle g(s_i)\rangle = 0$ for all i. Hence, using $\langle g(s_i)s_i\rangle = 1$ (see S2.1.1), Eq. (S8) is represented as $\delta_{ij}\delta_{kl} + \delta_{il}\delta_{jk}$ for $i \neq k$.

Case 2. For $i = k$, Eq. (S8) becomes

$$\langle g(s_i)^2\, s_j\, s_l + (E(\mathbf{s}) - E_0)\frac{\partial g(s_i)}{\partial s_i} s_j\, s_l\rangle. \quad \text{(S9)}$$

Case 2-1. When $i = k$ and $j \neq l$, Eq. (S9) becomes zero because either s_j or s_l is invariably independent on other variables and $(E(\mathbf{s}) - E_0)\partial g(s_i)/\partial s_i$ is an even function of $s_1, \ldots, s_N$, so that $(g(s_i)^2 s_j s_l + (E(\mathbf{s}) - E_0)\partial g(s_i)/\partial s_i s_j s_l)$ is definitely an odd function for s_j and s_l and its expectation is zero (see S2.1.2).

Case 2-2. When $i = k$ and $j = l$, Eq. (S9) becomes

$$\langle g(s_i)^2\, s_j^2 + (E(\mathbf{s}) - E_0)\frac{\partial g(s_i)}{\partial s_i} s_j^2\rangle$$

$$= \langle z_i'^2\, s_j^2\rangle + \sum_{m=1}^{N} \langle z_m\, z_i''\, s_j^2\rangle - (N\langle z_i\rangle + 1)\, \langle z_i''\, s_j^2\rangle, \quad \text{(S10)}$$

which is an even function of $s_1, \ldots, s_N$.

Case 2-2-1. When $i = k = j = l$, using the relationship of $\langle z_i'^2 s_i^2\rangle = \langle z_i'' s_i^2\rangle + 2$ (see S2.1.1), Eq. (S10) becomes

$$\langle z_i'^2 s_i^2 \rangle + (N-1)\langle z_i \rangle \langle z_i'' s_i^2 \rangle + \langle z_i z_i'' s_i^2 \rangle - (N\langle z_i \rangle + 1)\langle z_i'' s_i^2 \rangle$$
$$= \langle z_i'' s_i^2 \rangle + 2 + \langle z_i z_i'' s_i^2 \rangle - (\langle z_i \rangle + 1)\langle z_i'' s_i^2 \rangle$$
$$= 2 + \langle z_i z_i'' s_i^2 \rangle - \langle z_i \rangle \langle z_i'' s_i^2 \rangle$$
$$= 2 + \mathrm{cov}(z_i, z_i'' s_i^2). \quad \text{(S11)}$$

We define $\rho := \mathrm{cov}(z_i, z_i'' s_i^2)$.

Case 2-2-2. When $i = k \neq j = l$, using the relationship of $\langle z_i'^2 \rangle = \langle z_i'' \rangle$ (see S2.1.1), Eq. (S10) becomes

$$\langle z_i'' \rangle \langle s_i^2 \rangle + (N-2)\langle z_i \rangle \langle z_i'' \rangle \langle s_i^2 \rangle + \langle z_i z_i'' \rangle \langle s_i^2 \rangle + \langle z_i s_i^2 \rangle \langle z_i'' \rangle$$
$$- (N\langle z_i \rangle + 1)\langle z_i'' \rangle \langle s_i^2 \rangle$$
$$= \langle z_i z_i'' \rangle \langle s_i^2 \rangle + \langle z_i s_i^2 \rangle \langle z_i'' \rangle - 2\langle z_i \rangle \langle z_i'' \rangle \langle s_i^2 \rangle$$
$$= \mathrm{cov}(z_i, z_i'') \langle s_i^2 \rangle + \mathrm{cov}(z_i, s_i^2) \langle z_i'' \rangle. \quad \text{(S12)}$$

We define $\omega := \mathrm{cov}(z_i, z_i'') \langle s_i^2 \rangle + \mathrm{cov}(z_i, s_i^2) \langle z_i'' \rangle$.

Accordingly, d^2L is represented as

$$d^2L = \textstyle\sum_{i\neq k} (\delta_{ij}\,\delta_{kl} + \delta_{il}\,\delta_{jk})\, dK_{ij}\, dK_{kl} + \sum_{i=k=j=l} (2+\rho)\, dK_{ij}\, dK_{kl} + \sum_{i=k\neq j=l} \omega\, dK_{ij}\, dK_{kl}$$
$$= \textstyle\sum_{i\neq k} (dK_{ii}\, dK_{kk} + dK_{ik}\, dK_{ki}) + \sum_i (2+\rho)\, dK_{ii}^2 + \sum_{i\neq j} \omega\, dK_{ij}^2$$
$$= \textstyle\sum_i (1+\rho)\, dK_{ii}^2 + \sum_i \sum_k dK_{ii}\, dK_{kk} + \sum_{i\neq j} (\omega\, dK_{ij}^2 + dK_{ij}\, dK_{ji})$$
$$= \textstyle\sum_i (1+\rho)\, dK_{ii}^2 + (\sum_i dK_{ii})^2 + 1/2 \cdot \sum_{i\neq j} (\omega dK_{ij}^2 + 2dK_{ij}\, dK_{ji} + \omega dK_{ji}^2). \quad \text{(S13)}$$

From a condition where a discriminant of a quadratic equation in the third term is negative, all coefficients of d^2L are non-negative if $\rho > -1$, $\omega > 0$, and $1 - \omega^2 < 0$ hold. Therefore, the necessary and sufficient conditions where $W = A^{-1}$ becomes a stable equilibrium point are $\rho > -1$ and $\omega > 1$.

S2.3 The absence of spurious solutions of the EGHR with Gaussian sources

In this section, we show the absence of spurious solutions of the EGHR if sources obey a Gaussian distribution. Note that, while ICA is not defined for Gaussian sources, the EGHR can still perform input whitening. Furthermore, we show that there is no spurious solution as long as the source distribution is nearly Gaussian in the next section.

Assuming that s_i obeys a normal Gaussian distribution, $p_0(s_i) = N(s_i; \mu = 0, \sigma^2 = 1)$, z_i, z_i', z_i'', and E_0 are calculated as $z_i = u_i^2/2 + 1/2 \cdot \log 2\pi$, $z_i' = g(u_i) = u_i$, $z_i'' = 1$, and $E_0 = N$

$\langle s_i^2/2 + 1/2\cdot\log 2\pi\rangle + 1 = N/2 + N/2\cdot\log 2\pi + 1$, respectively. Then $\dot{K}$ can be rewritten as

$$\dot{K} \propto -\langle (E(\mathbf{u}) - E_0)\, \mathbf{u}\, \mathbf{s}^T\rangle = -K \langle (\textstyle\sum_k z_k - E_0)\, \mathbf{s}\, \mathbf{s}^T\rangle$$
$$= -K \langle (1/2 \cdot \textstyle\sum_k u_k^2 - N/2 - 1)\, \mathbf{s}\, \mathbf{s}^T\rangle. \quad \text{(S14)}$$

Here, $\sum_k u_k^2$ is calculated as $\sum_k u_k^2 = \sum_k (\sum_l K_{kl} s_l)^2 = \sum_k (\sum_l K_{kl}^2 s_l^2 + \sum_{l\neq m} K_{kl} K_{km} s_l s_m)$. When $i = j$, $\langle (1/2\cdot\sum_k u_k^2 - N/2 - 1) s_i s_j\rangle$ is calculated as

$$\langle (1/2 \cdot \textstyle\sum_k \sum_l K_{kl}^2\, s_l^2 - N/2 - 1)\, s_i^2\rangle$$
$$= \langle 1/2 \cdot \textstyle\sum_k K_{ki}^2\, s_i^4\rangle + \langle 1/2 \cdot \textstyle\sum_k \sum_{l\neq i} K_{kl}^2\, s_l^2\, s_i^2\rangle - (N/2 + 1)\, \langle s_i^2\rangle$$
$$= 3/2 \cdot \textstyle\sum_k K_{ki}^2 + 1/2 \cdot \textstyle\sum_k \sum_{l\neq i} K_{kl}^2 - (N/2 + 1)$$
$$= \textstyle\sum_k K_{ki}^2 + 1/2 \cdot \textstyle\sum_k \sum_l K_{kl}^2 - (N/2 + 1). \quad \text{(S15)}$$

The condition where Eq. (S15) becomes zero for all i is $\sum_k K_{ki}^2 = 1$ for all i. On the other hand, when $i \neq j$, $\langle (1/2\cdot\sum_k u_k^2 - N/2 - 1)\, s_i\, s_j\rangle$ is calculated as

$$\langle 1/2 \cdot \textstyle\sum_k \sum_{l\neq m} K_{kl}\, K_{km}\, s_l\, s_m\, s_i\, s_j\rangle$$
$$= \langle 1/2 \cdot \textstyle\sum_k (K_{ki}\, K_{kj}\, s_i\, s_j\, s_i\, s_j + K_{kj}\, K_{ki}\, s_j\, s_i\, s_i\, s_j)\rangle$$
$$= \textstyle\sum_k K_{ki}\, K_{kj}. \quad \text{(S16)}$$

The condition where Eq. (S16) becomes zero for all i, j ($i \neq j$) is $\sum_k K_{ki}\, K_{kj} = 0$ for all i, j ($i \neq j$). Taken together, Eq. (S14) is rewritten as

$$\dot{K} \propto -K\, (K^T K + 1/2\ \mathrm{tr}(K^T K)\, I - (N/2 + 1)\, I)$$
$$= -K\, K^T K - 1/2\ \mathrm{tr}(K^T K)\, K + (N/2 + 1)\, K$$
$$= -(K\, K^T - I)\, K - 1/2\ (\mathrm{tr}(K^T K) - N)\, K. \quad \text{(S17)}$$

The first term of Eq. (S17) helps to decorrelate the elements of $\mathbf{u}$, while the second term only scales $\mathbf{u}$. Therefore, the condition of $\dot{K} = 0$ is $K^T K = I$ or $K = 0$. It is easy to see that $K = 0$ is an unstable equilibrium point from Eq. (S17). On the other hand, the $K^T K = I$ solution indicates that K is a rotation matrix R. From an analysis of the linear stability, it turns out that $K = R$ resides at a valley of L, and $K = 0$ resides at a peak of L (see S2.2). There is no other peak or valley of L. Therefore, the global minimum of L is given when $K = R$, with which input whitening is achieved.

S2.4 The absence of spurious solutions of the EGHR with the non-Gaussian sources with small non-Gaussianity

In this section, we proposed an approach to calculate the curvature of L when the sources obey a probability distribution similar to Gaussian distribution. We show that there is no spurious solution if the sources are distributed close to a Gaussian distribution.

Step 1. We define the general form of a source distribution by $p_0(s_i) \propto \exp(\gamma_1 s_i^2 + \gamma_2 s_i^4 + \gamma_3 s_i^6 + \cdots)$. Assuming that $\gamma_1 = -1/2$, $\gamma_2 = \varepsilon$, and γ_3, γ_4, … are order $O(\varepsilon^2)$ or less, where ε is a small constant, we obtain the first order approximation of $p_0(s_i)$ with ε as

$$p_0(s_i) \propto \exp(-s_i^2/2)\,(1 + \varepsilon\, s_i^4)$$
$$\propto N(s_i;\, 0,\, 1)\,(1 + \varepsilon\, s_i^4). \qquad \text{(S18)}$$

The integral of $N(s_i; 0, 1)(1 + \varepsilon s_i^4)$ can be regarded as the expectation of $(1 + \varepsilon s_i^4)$ over $N(s_i; 0, 1)$, which is calculated as

$$Z = \int N(s_i;\, 0,\, 1)\,(1 + \varepsilon\, s_i^4)\, ds_i = \langle 1 + \varepsilon\, s_i^4 \rangle_{N(si)} = 1 + 3\,\varepsilon, \qquad \text{(S19)}$$

where $\langle \bullet \rangle_{N(si)}$ is the expectation over $N(s_i; 0, 1)$. Thus, $p_0(s_i)$ can be further approximated as

$$p_0(s_i) = N(s_i;\, 0,\, 1)\frac{1 + \varepsilon s_i^4}{1 + 3\varepsilon} \approx N(s_i;\, 0,\, 1)\,(1 + \varepsilon\, s_i^4 - 3\,\varepsilon). \qquad \text{(S20)}$$

The kurtosis of $p_0(s_i)$ is calculated as $\langle s_i^4 \rangle / \langle s_i^2 \rangle^2 - 3 = 24\varepsilon$; therefore, $p_0(s_i)$ will be a super-Gaussian distribution if ε is positive, while it will be sub-Gaussian if ε is negative. We then consider $z(u_i)$ for the general prior $p_0(s_i)$ as $z(u_i) = -\log p_0(u_i)$. We assume that $z(u_i)$ is an even function. The derivative is represented as $g(u_i) = z'(u_i)$. In addition, we define $\varphi(\mathbf{s}) = \prod_k (1 + \varepsilon s_k^4 - 3\varepsilon) \approx 1 + \varepsilon\,(\sum_k s_k^4 - 3N)$, which indicates the difference of $p_0(\mathbf{s})$ from $N(\mathbf{s}; \mathbf{0}, I)$. Using $K = WA$ and $\varphi(\mathbf{s})$, Eq. (1) is rewritten as

$$\dot{K} \propto -\langle (E(\mathbf{u}) - E_0)\, g(\mathbf{u})\, \mathbf{s}^T \rangle_{p0(\mathbf{s})}\, A^T A$$
$$= -\langle (E(\mathbf{u}) - E_0)\, g(\mathbf{u})\, \mathbf{s}^T\, \varphi(\mathbf{s}) \rangle_{N(\mathbf{s})}\, A^T A$$
$$= -\langle (E(\mathbf{u}) - E_0)\, g(\mathbf{u})\, \mathbf{u}^T\, \varphi(\mathbf{s}) \rangle_{N(\mathbf{u})}\, K^{-T} A^T A. \qquad \text{(S21)}$$

From the relationship of $\mathbf{s} = K^{-1}\mathbf{u}$, $\varphi(\mathbf{s})$ is expanded as

$$\varphi(\mathbf{s}) = 1 + \varepsilon\, \{\textstyle\sum_k (\sum_l (K^{-1})_{kl}\, u_l)^4 - 3N\}$$
$$= 1 + \varepsilon\, [\textstyle\sum_k \{\sum_l (K^{-1})_{kl}^4\, u_l^4 + \sum_{l\neq m} (K^{-1})_{kl}^3\, u_l^3\, (K^{-1})_{km}\, u_m$$
$$+ \textstyle\sum_{l\neq m} (K^{-1})_{kl}^2\, u_l^2\, (K^{-1})_{km}^2\, u_m^2 + \cdots\} - 3N]. \qquad \text{(S22)}$$

We define a matrix C by $C := \langle (E(\mathbf{u}) - E_0) g(\mathbf{u}) \mathbf{u}^T \varphi(\mathbf{s}) \rangle_{N(\mathbf{u})}$. If and only if C is zero, $\dot{K}$ becomes zero. Because $E(\mathbf{u})$ is an even function, the diagonal elements of C become

$$\begin{aligned} C_{ii} &= \langle (E(\mathbf{u}) - E_0)\, g(u_i)\, u_i \\ &\quad \cdot [1 - 3N\varepsilon + \varepsilon \textstyle\sum_k \{\sum_l (K^{-1})_{kl}^4\, u_l^4 + \sum_{l \neq m} (K^{-1})_{kl}^2\, u_l^2\, (K^{-1})_{km}^2\, u_m^2\}] \rangle_{N(\mathbf{u})} \\ &= (1 - 3N\varepsilon) \langle (E(\mathbf{u}) - E_0)\, g(u_i)\, u_i \rangle_{N(\mathbf{u})} + \varepsilon \langle (E(\mathbf{u}) - E_0)\, g(u_i)\, u_i \textstyle\sum_k \{\sum_l (K^{-1})_{kl}^4\, u_l^4 \\ &\quad + \textstyle\sum_{l \neq m} (K^{-1})_{kl}^2\, u_l^2\, (K^{-1})_{km}^2\, u_m^2\} \rangle_{N(\mathbf{u})}, \end{aligned} \tag{S23}$$

and its non-diagonal elements ($i \neq j$) are

$$\begin{aligned} C_{ij} &= \langle (E(\mathbf{u}) - E_0)\, g(u_i)\, u_j \cdot [1 - 3N\varepsilon + \varepsilon \textstyle\sum_k \{\sum_{l \neq m} (K^{-1})_{kl}^3\, u_l^3\, (K^{-1})_{km}\, u_m\}] \rangle_{N(\mathbf{u})} \\ &= (1 - 3N\varepsilon) \langle (E(\mathbf{u}) - E_0)\, g(u_i)\, u_j \rangle_{N(\mathbf{u})} \\ &\quad + \varepsilon \langle (E(\mathbf{u}) - E_0)\, g(u_i)\, u_j \cdot \textstyle\sum_k \{(K^{-1})_{ki}^3\, u_i^3\, (K^{-1})_{kj}\, u_j + (K^{-1})_{kj}^3\, u_j^3\, (K^{-1})_{ki}\, u_i\} \rangle_{N(\mathbf{u})} \\ &= (1 - 3N\varepsilon) \langle (E(\mathbf{u}) - E_0)\, g(u_i)\, u_j \rangle_{N(\mathbf{u})} \\ &\quad + \varepsilon \textstyle\sum_k \{c_1\, (K^{-1})_{ki}^3\, (K^{-1})_{kj} + c_2\, (K^{-1})_{kj}^3\, (K^{-1})_{ki}\}, \end{aligned} \tag{S24}$$

where we set $c_1 = \langle (E(\mathbf{u}) - E_0) g(u_i) u_i^3 u_j^2 \rangle_{N(\mathbf{u})}$ and $c_2 = \langle (E(\mathbf{u}) - E_0) g(u_i) u_i u_j^4 \rangle_{N(\mathbf{u})}$. When we assume the covariance matrix of $\mathbf{u}$, $\langle \mathbf{u}\mathbf{u}^T \rangle$, is a diagonal, $\langle (E(\mathbf{u}) - E_0) g(u_i) u_j \rangle_{N(\mathbf{u})}$ becomes zero. In this condition, C_{ij} becomes zero if and only if $\sum_k \{c_1\, (K^{-1})_{ki}^3\, (K^{-1})_{kj} + c_2\, (K^{-1})_{kj}^3\, (K^{-1})_{ki}\} = 0$ holds for all $i \neq j$. Whereas, if u_i and u_j are correlated, $\langle (E(\mathbf{u}) - E_0)\, g(u_i)\, u_j \rangle_{N(\mathbf{u})}$ is not equal to zero; therefore, $\mathbf{u}$ must be decorrelated in order for C_{ij} to be zero. Accordingly, because $\mathbf{u}$ should obey a normal Gaussian distribution to reach an equilibrium point, a necessary condition for $\dot{K} = 0$ is that K becomes proportional to a rotation matrix R.

Step 2. In this step, we assume K is closed to a rotation matrix ($K = R$) and determine the optimal rotations that gives $\dot{K} = 0$. Note that $N(\mathbf{u}; \mathbf{0}, I) = N(\mathbf{s}; \mathbf{0}, I)$ holds in this case because rotation of a whitened Gaussian distribution is also white. In the following, we first consider the case where A is a rotation matrix, and later generalize the results to non-rotational A. Moreover, we assume that E_0 satisfies

$$0 = (1 - 3N\varepsilon) \langle (E(\mathbf{u}) - E_0)\, g(u_i)\, u_i \rangle_{N(\mathbf{u})} + \varepsilon \langle (E(\mathbf{u}) - E_0)\, g(u_i)\, u_i \textstyle\sum_l u_l^4 \rangle_{N(\mathbf{u})}. \tag{S25}$$

In this case, Eq. (S21) is rewritten as $\dot{K} \propto -\mathbf{E}[C\, K]$, where $\mathbf{E}[\bullet]$ indicates temporal averaging of $\bullet$ with averaging time constant $> \tau_W$. Importantly, although K deviates from a rotation matrix because $K + \dot{K}\Delta t$ is not always a rotation matrix, its average $\mathbf{E}[K]$ continues to be a rotation matrix (see S2.3). Since we suppose $\mathbf{E}[K]$ to be a rotation

matrix, the degree of freedom of $\mathbf{E}[K]$ is $N(N-1)/2$. We define a rotation in an s_i–s_j plane to be θ_{ij} ($1 \le i < j \le N$) and the corresponding rotation matrix as $R^{\theta ij}$. Here, $R^{\theta ij}$ is the same as the identity matrix I except that the (i, i)th, (i, j)th, (j, i)th, and (j, j)th elements are $\cos\theta_{ij}$, $-\sin\theta_{ij}$, $\sin\theta_{ij}$, and $\cos\theta_{ij}$, respectively. Therefore, any rotation can be written as the product of various $R^{\theta ij}$ for any pair of axes. Let us suppose $K = R^{\theta ij}B^{\theta ij}$ holds, where $B^{\theta ij}$ is any rotation matrix except a rotation in an s_i–s_j plane. Then, $\dot{R}^{\theta ij}$ can be written as

$$\dot{R}^{\theta ij} = \dot{K}\,(B^{\theta ij})^{-1} \propto -\mathbf{E}[C\,R^{\theta ij}], \tag{S26}$$

and Eq. (S26) is approximated as

$$\dot{R}^{\theta ij}\,R^{\theta ijT} \propto -\mathbf{E}[C]. \tag{S27}$$

The elements of $\dot{R}^{\theta ij}$ except for those of (i, i), (i, j), (j, i), and (j, j) are fixed to be zero. Since $\dot{R}^{\theta ij}{}_{ii} = -\sin\theta_{ij}\dot{\theta}_{ij}$, $\dot{R}^{\theta ij}{}_{ij} = -\cos\theta_{ij}\dot{\theta}_{ij}$, $\dot{R}^{\theta ij}{}_{ji} = \cos\theta_{ij}\dot{\theta}_{ij}$, and $\dot{R}^{\theta ij}{}_{jj} = -\sin\theta_{ij}\dot{\theta}_{ij}$ hold, the (i, i)th, (i, j)th, (j, i)th, and (j, j)th elements of $\dot{R}^{\theta ij}\,R^{\theta ijT}$ are respectively given by

$$\begin{aligned}
(\dot{R}^{\theta ij}\,R^{\theta ijT})_{ii} &= \textstyle\sum_k \dot{R}^{\theta ij}{}_{ik}\,R^{\theta ij}{}_{ik} = -\sin\theta_{ij}\dot{\theta}_{ij}\cos\theta_{ij} + (-\cos\theta_{ij}\dot{\theta}_{ij})\,(-\sin\theta_{ij}) = 0,\\
(\dot{R}^{\theta ij}\,R^{\theta ijT})_{ij} &= \textstyle\sum_k \dot{R}^{\theta ij}{}_{ik}\,R^{\theta ij}{}_{jk} = -\sin\theta_{ij}\dot{\theta}_{ij}\sin\theta_{ij} + (-\cos\theta_{ij}\dot{\theta}_{ij})\cos\theta_{ij} = -\dot{\theta}_{ij},\\
(\dot{R}^{\theta ij}\,R^{\theta ijT})_{ji} &= \textstyle\sum_k \dot{R}^{\theta ij}{}_{jk}\,R^{\theta ij}{}_{ik} = \cos\theta_{ij}\dot{\theta}_{ij}\cos\theta_{ij} + (-\sin\theta_{ij}\dot{\theta}_{ij})\,(-\sin\theta_{ij}) = \dot{\theta}_{ij},\\
(\dot{R}^{\theta ij}\,R^{\theta ijT})_{jj} &= \textstyle\sum_k \dot{R}^{\theta ij}{}_{jk}\,R^{\theta ij}{}_{jk} = \cos\theta_{ij}\dot{\theta}_{ij}\sin\theta_{ij} + (-\sin\theta_{ij}\dot{\theta}_{ij})\cos\theta_{ij} = 0.
\end{aligned} \tag{S28}$$

Thus, from Eqs. (S27) and (S28), we obtain

$$\begin{aligned}
\mathbf{E}[C_{ij}] &= \mathbf{E}[\varepsilon\textstyle\sum_k(c_1K_{ik}{}^3K_{jk} + c_2K_{ik}K_{jk}{}^3)] \propto \dot{\theta}_{ij},\\
\mathbf{E}[C_{ji}] &= \mathbf{E}[\varepsilon\textstyle\sum_k(c_1K_{ik}K_{jk}{}^3 + c_2K_{ik}{}^3K_{jk})] \propto -\dot{\theta}_{ij}.
\end{aligned} \tag{S29}$$

From Eq. (S29), $\mathbf{E}[\sum_k K_{ik}{}^3K_{jk}] = -\mathbf{E}[\sum_k K_{ik}K_{jk}{}^3]$ holds. Therefore, we obtain

$$\begin{aligned}
&\dot{\theta}_{ij} \propto \varepsilon(c_1 - c_2)\mathbf{E}[\textstyle\sum_k K_{ik}{}^3\,K_{jk}] \text{ and}\\
&\dot{\theta}_{ij} \propto -\varepsilon(c_1 - c_2)\mathbf{E}[\textstyle\sum_k K_{ik}\,K_{jk}{}^3].
\end{aligned} \tag{S30}$$

Then, K_{ik} and K_{jk} can be expanded as

$$\begin{aligned}
K_{ik} &= \textstyle\sum_l R^{\theta ij}{}_{il}\,B^{\theta ij}{}_{lk} = \cos\theta_{ij}\,B^{\theta ij}{}_{ik} - \sin\theta_{ij}\,B^{\theta ij}{}_{jk},\\
K_{jk} &= \textstyle\sum_l R^{\theta ij}{}_{jl}\,B^{\theta ij}{}_{lk} = \sin\theta_{ij}\,B^{\theta ij}{}_{ik} + \cos\theta_{ij}\,B^{\theta ij}{}_{jk},
\end{aligned} \tag{S31}$$

and $K_{ik}{}^3K_{jk}$ and $K_{jk}{}^3\,K_{ik}$ can be calculated as

$$K_{ik}^3\, K_{jk} = (\cos\theta_{ij}\, B^{\theta ij}{}_{ik} - \sin\theta_{ij}\, B^{\theta ij}{}_{jk})^3\, (\sin\theta_{ij}\, B^{\theta ij}{}_{ik} + \cos\theta_{ij}\, B^{\theta ij}{}_{jk})$$
$$= (\cos^2\theta_{ij}\, B^{\theta ij}{}_{ik}{}^2 - 2\sin\theta_{ij}\cos\theta_{ij}\, B^{\theta ij}{}_{ik}\, B^{\theta ij}{}_{jk} + \sin^2\theta_{ij}\, B^{\theta ij}{}_{jk}{}^2)$$
$$\cdot\, (\sin\theta_{ij}\cos\theta_{ij}\, (B^{\theta ij}{}_{ik}{}^2 - B^{\theta ij}{}_{jk}{}^2) + (\cos^2\theta_{ij} - \sin^2\theta_{ij})\, B^{\theta ij}{}_{ik}\, B^{\theta ij}{}_{jk}),$$
$$K_{jk}^3\, K_{ik} = (\sin\theta_{ij}\, B^{\theta ij}{}_{ik} + \cos\theta_{ij}\, B^{\theta ij}{}_{jk})^3\, (\cos\theta_{ij}\, B^{\theta ij}{}_{ik} - \sin\theta_{ij}\, B^{\theta ij}{}_{jk})$$
$$= (\sin^2\theta_{ij}\, B^{\theta ij}{}_{ik}{}^2 + 2\sin\theta_{ij}\cos\theta_{ij}\, B^{\theta ij}{}_{ik}\, B^{\theta ij}{}_{jk} + \cos^2\theta_{ij}\, B^{\theta ij}{}_{jk}{}^2)$$
$$\cdot\, (\sin\theta_{ij}\cos\theta_{ij}\, (B^{\theta ij}{}_{ik}{}^2 - B^{\theta ij}{}_{jk}{}^2) + (\cos^2\theta_{ij} - \sin^2\theta_{ij})\, B^{\theta ij}{}_{ik}\, B^{\theta ij}{}_{jk}). \qquad \text{(S32)}$$

Because $\theta_{ij} = 0, \pi/2$ are sufficient conditions for K to be an ICA solution, by substituting $\theta_{ij} = 0, \pi/2$ into Eq. (S30), we obtain

$$\dot{\theta}_{ij}|_{\theta ij=0} \propto \varepsilon(c_2 - c_1)\mathbf{E}[\textstyle\sum_k B^{\theta ij}{}_{ik}{}^3\, B^{\theta ij}{}_{jk}] = 0,$$
$$\dot{\theta}_{ij}|_{\theta ij=\pi/2} \propto \varepsilon(c_2 - c_1)\mathbf{E}[\textstyle\sum_k B^{\theta ij}{}_{ik}\, B^{\theta ij}{}_{jk}{}^3] = 0. \qquad \text{(S33)}$$

Therefore, $\mathbf{E}[\sum_k K_{ik}^3 K_{jk}]$ and $\mathbf{E}[\sum_k K_{jk}^3 K_{ik}]$ are calculated as

$$\mathbf{E}[\textstyle\sum_k K_{ik}^3\, K_{jk}] = \mathbf{E}[\sum_k\{(\cos^2\theta_{ij}\, B^{\theta ij}{}_{ik}{}^2 + \sin^2\theta_{ij}\, B^{\theta ij}{}_{jk}{}^2)\sin\theta_{ij}\cos\theta_{ij}\, (B^{\theta ij}{}_{ik}{}^2 - B^{\theta ij}{}_{jk}{}^2)$$
$$- 2\sin\theta_{ij}\cos\theta_{ij}\, B^{\theta ij}{}_{ik}\, B^{\theta ij}{}_{jk}\, (\cos^2\theta_{ij} - \sin^2\theta_{ij})\, B^{\theta ij}{}_{ik}\, B^{\theta ij}{}_{jk}\}]$$
$$= \mathbf{E}[\sin\theta_{ij}\cos\theta_{ij} \textstyle\sum_k \{(\cos^2\theta_{ij}\, B^{\theta ij}{}_{ik}{}^2 + \sin^2\theta_{ij}\, B^{\theta ij}{}_{jk}{}^2)\, (B^{\theta ij}{}_{ik}{}^2 - B^{\theta ij}{}_{jk}{}^2)$$
$$- 2B^{\theta ij}{}_{ik}{}^2\, B^{\theta ij}{}_{jk}{}^2\, (\cos^2\theta_{ij} - \sin^2\theta_{ij})\}]$$
$$= \frac{1}{4}\mathbf{E}[\sin 2\theta_{ij} \textstyle\sum_k\{((1+\cos 2\theta_{ij})\, B^{\theta ij}{}_{ik}{}^2 + (1-\cos 2\theta_{ij})\, B^{\theta ij}{}_{jk}{}^2)\, (B^{\theta ij}{}_{ik}{}^2 - B^{\theta ij}{}_{jk}{}^2)$$
$$- 4B^{\theta ij}{}_{ik}{}^2\, B^{\theta ij}{}_{jk}{}^2 \cos 2\theta_{ij}\}]$$
$$= \frac{1}{4}\mathbf{E}[\sin 2\theta_{ij}\textstyle\sum_k\{(B^{\theta ij}{}_{ik}{}^4 - 6B^{\theta ij}{}_{ik}{}^2 B^{\theta ij}{}_{jk}{}^2 + B^{\theta ij}{}_{jk}{}^4)\cos 2\theta_{ij} + B^{\theta ij}{}_{ik}{}^4 - B^{\theta ij}{}_{jk}{}^4\}],$$
$$\mathbf{E}[\textstyle\sum_k K_{jk}^3\, K_{ik}] = \mathbf{E}[\sum_k \{(\sin^2\theta_{ij}\, B^{\theta ij}{}_{ik}{}^2 + \cos^2\theta_{ij}\, B^{\theta ij}{}_{jk}{}^2)\sin\theta_{ij}\cos\theta_{ij}\, (B^{\theta ij}{}_{ik}{}^2 - B^{\theta ij}{}_{jk}{}^2)$$
$$+ 2\sin\theta_{ij}\cos\theta_{ij}\, B^{\theta ij}{}_{ik}\, B^{\theta ij}{}_{jk}\, (\cos^2\theta_{ij} - \sin^2\theta_{ij})\, B^{\theta ij}{}_{ik}\, B^{\theta ij}{}_{jk}\}]$$
$$= \mathbf{E}[\sin\theta_{ij}\cos\theta_{ij} \textstyle\sum_k \{(\sin^2\theta_{ij}\, B^{\theta ij}{}_{ik}{}^2 + \cos^2\theta_{ij}\, B^{\theta ij}{}_{jk}{}^2)\, (B^{\theta ij}{}_{ik}{}^2 - B^{\theta ij}{}_{jk}{}^2)$$
$$+ 2B^{\theta ij}{}_{ik}{}^2\, B^{\theta ij}{}_{jk}{}^2\, (\cos^2\theta_{ij} - \sin^2\theta_{ij})\}]$$
$$= \frac{1}{4}\mathbf{E}[\sin 2\theta_{ij} \textstyle\sum_k \{((1-\cos 2\theta_{ij})\, B^{\theta ij}{}_{ik}{}^2 + (1+\cos 2\theta_{ij})\, B^{\theta ij}{}_{jk}{}^2)\, (B^{\theta ij}{}_{ik}{}^2 - B^{\theta ij}{}_{jk}{}^2)$$
$$+ 4B^{\theta ij}{}_{ik}{}^2\, B^{\theta ij}{}_{jk}{}^2 \cos 2\theta_{ij}\}]$$
$$= \frac{1}{4}\mathbf{E}[\sin 2\theta_{ij}\textstyle\sum_k\{(-B^{\theta ij}{}_{ik}{}^4 + 6B^{\theta ij}{}_{ik}{}^2 B^{\theta ij}{}_{jk}{}^2 - B^{\theta ij}{}_{jk}{}^4)\cos 2\theta_{ij} + B^{\theta ij}{}_{ik}{}^4 - B^{\theta ij}{}_{jk}{}^4\}]. \qquad \text{(S34)}$$

From Eq. (S34), $\mathbf{E}[B^{\theta ij}{}_{ik}{}^4] = \mathbf{E}[B^{\theta ij}{}_{jk}{}^4]$ hold since $\mathbf{E}[\sum_k K_{ik}^3 K_{jk}] = -\mathbf{E}[\sum_k K_{ik} K_{jk}^3]$. Consequently, from Eq. (S30), we obtain

$$\dot{\theta}_{ij} \propto \frac{1}{4}\varepsilon(c_1 - c_2)\mathbf{E}[\sin 2\theta_{ij}\cos 2\theta_{ij}\textstyle\sum_k({B^{\theta ij}_{ik}}^4 - 6{B^{\theta ij}_{ik}}^2{B^{\theta ij}_{jk}}^2 + {B^{\theta ij}_{jk}}^4)]$$

$$= \frac{1}{8}\varepsilon(c_1 - c_2)\mathbf{E}[\sin 4\theta_{ij}\textstyle\sum_k({B^{\theta ij}_{ik}}^4 - 6{B^{\theta ij}_{ik}}^2{B^{\theta ij}_{jk}}^2 + {B^{\theta ij}_{jk}}^4)]. \quad \text{(S35)}$$

By assuming θ_{ij} is independent of $B^{\theta ij}$, Eq. (S35) can be approximated as

$$\dot{\theta}_{ij} \propto \frac{1}{8}\varepsilon(c_1 - c_2)\sin 4\theta_{ij}\ \mathbf{E}[\textstyle\sum_k({B^{\theta ij}_{ik}}^4 - 6{B^{\theta ij}_{ik}}^2{B^{\theta ij}_{jk}}^2 + {B^{\theta ij}_{jk}}^4)]. \quad \text{(S36)}$$

Numerical calculations suggest that $\mathbf{E}[\sum_k({B^{\theta ij}_{ik}}^4 - 6{B^{\theta ij}_{ik}}^2{B^{\theta ij}_{jk}}^2 + {B^{\theta ij}_{jk}}^4)]$ is positive-definite when K is a rotation matrix. Therefore, if and only if $\theta_{ij} = k\pi/4$ ($k = 0, 1, \ldots, 7$), $\dot{\theta}_{ij}$ becomes zero. Notably, if $\theta_{ij} = 0$ is stable $\theta_{ij} = k\pi/4$ ($k = 2, 4, 6$) are also stable, while $\theta_{ij} = k\pi/4$ ($k = 1, 3, 5, 7$) are unstable. If $\theta_{ij} = 0$ is unstable, the exactly opposite occurs. Therefore, if $\theta_{ij} = 0$ is stable, which depends on the sign of ε $(c_1 - c_2)$, only ICA solutions give equilibrium points of θ_{ij}s, which means that there is no spurious solution of EGHR for the source distribution near Gaussian. Furthermore, for any transform matrix A, A^TA is a positive definite matrix, so that even when A is not a rotation matrix, there is no spurious solution of EGHR for the source distribution near Gaussian.

Step 3. Lastly, we evaluate the sign of $\varepsilon(c_1 - c_2)$ to determine whether these ICA solutions are stable or unstable. Indeed, the sign of $(c_1 - c_2) = \langle(E(\mathbf{u}) - E_0)g(u_i)(u_i^3\, u_j^2 - u_i\, u_j^4)\rangle_{N(\mathbf{u})}$ depends on the form of $z(u_i)$. If we assume $z(u_i) = u_i^2/2 - \delta u_i^4$, $g(u_i)$ is calculated $g(u_i) = u_i - 4\delta u_i^3$ and E_0 can be supposed as $E_0 = N/2 + 1 + O(\delta) + O(\varepsilon)$, where $O(\delta)$ and $O(\varepsilon)$ are functions of δ and ε, respectively. Because of the symmetry of indexes i and j, $\langle(E(\mathbf{u}) - E_0)\,(u_i^4\, u_j^2 - u_i^2\, u_j^4)\rangle_{N(\mathbf{u})}$ is calculated as zero. Therefore, $(c_1 - c_2)$ is calculated as

$$\begin{aligned}(c_1 - c_2) &= \langle(E(\mathbf{u}) - E_0)g(u_i)(u_i^3\, u_j^2 - u_i\, u_j^4)\rangle_{N(\mathbf{u})}\\ &= \langle\{\textstyle\sum_m (u_m^2/2 - \delta u_m^4) - N/2 - 1 - O(\delta) - O(\varepsilon)\}\,(u_i - 4\delta u_i^3)\,(u_i^3\, u_j^2 - u_i\, u_j^4)\rangle_{N(\mathbf{u})}\\ &= -4\delta\langle\{\textstyle\sum_m (u_m^2/2 - \delta u_m^4) - N/2 - 1 - O(\delta) - O(\varepsilon)\}\,(u_i^6\, u_j^2 - u_i^4\, u_j^4)\rangle_{N(\mathbf{u})}. \quad \text{(S37)}\end{aligned}$$

If we assume δ is small, Eq. (S37) is approximated as

$$\begin{aligned}(c_1 - c_2) &= -2\delta\langle(\textstyle\sum_m u_m^2 - N - 2)\,(u_i^6\, u_j^2 - u_i^4\, u_j^4)\rangle_{N(\mathbf{u})} + \delta(O(\delta)+O(\varepsilon))\\ &= -2\delta\{\langle u_i^8\, u_j^2 - u_i^6\, u_j^4\rangle_{N(\mathbf{u})} + \langle u_i^6\, u_j^4 - u_i^4\, u_j^6\rangle_{N(\mathbf{u})}\\ &\quad + (N-2)\langle u_m^2\rangle_{N(\mathbf{u})}\langle u_i^6u_j^2 - u_i^4u_j^4\rangle_{N(\mathbf{u})} - (N-2)\langle u_i^6u_j^2 - u_i^4u_j^4\rangle_{N(\mathbf{u})}\} + \delta(O(\delta)+O(\varepsilon)).\end{aligned}$$

(S38)

From S2.1.3, we get $\langle u_i^4 \rangle_{N(\mathbf{u})} = 3\langle u_i^2 \rangle_{N(\mathbf{u})} = 3$, $\langle u_i^6 \rangle_{N(\mathbf{u})} = 5\langle u_i^4 \rangle_{N(\mathbf{u})} = 15$, and , $\langle u_i^8 \rangle_{N(\mathbf{u})} = 7\langle u_i^6 \rangle_{N(\mathbf{u})} = 105$. Therefore, since $i \neq j$, we obtain

$$\begin{aligned}(c_1 - c_2) &= -2\delta\{105 - 15\cdot 3 + 15\cdot 3 - 3\cdot 15 \\ &\quad + (N-2)(15 - 3\cdot 3) - (N-2)(15 - 3\cdot 3)\} + \delta(O(\delta)+O(\varepsilon)) \\ &= -120\delta + \delta(O(\delta)+O(\varepsilon)).\end{aligned} \tag{S39}$$

Accordingly, from Eqs. (S36) and (S39), we obtain

$$\dot{\theta}_{ij} \propto -15\delta\varepsilon \sin 4\theta_{ij}\, \mathbf{E}[\textstyle\sum_k (B^{\theta ij}{}_{ik}{}^4 - 6B^{\theta ij}{}_{ik}{}^2 B^{\theta ij}{}_{jk}{}^2 + B^{\theta ij}{}_{jk}{}^4)]. \tag{S40}$$

Because $\delta\varepsilon$ can be a positive definite scalar if we choose so and $A^T A$ is a positive definite matrix, Eq. (S40) always converges to $K = I$ or its permutations or sign-flips, i.e., these are the only stable equilibrium point and there is no other stable equilibrium point on $K = R$. Taken together with the result from S2.3, we conclude that $K = I$ is the only stable equilibrium point and there is no other stable equilibrium point.

S2.5 Simplification of conventional local ICA rules

In this section, to speed up numerical simulations in Fig. 3B, we analytically simplify the conventional local ICA rules, assuming that A and W are rotation matrices.

S2.5.1 The Linsker rule

In the Linsker rule [20] (see also Eq. (5)), the dynamics of the inner states $\mathbf{v}$ is represented as $\tau_v \dot{\mathbf{v}} = -\mathbf{v} + \mathbf{u} + Q\mathbf{v}$, where $\mathbf{u} = W\mathbf{x}$ holds. If we assume that a change of $\mathbf{x}$ (therefore, a change of sources) is sufficiently slower than that of $\mathbf{v}$, $\dot{\mathbf{v}}$ converges to $\mathbf{0}$ before $\mathbf{x}$ changes. An equilibrium state of $\mathbf{v}$ can then be rewritten as $-\mathbf{v} + \mathbf{u} + Q\mathbf{v} = \mathbf{0}$ and solved as $\mathbf{v} = (I - Q)^{-1} W\mathbf{x}$. In the equilibrium state, $\langle \mathbf{v}\mathbf{x}^T \rangle = \langle (I - Q)^{-1} W\mathbf{x}\mathbf{x}^T \rangle = \langle (I - Q)^{-1} W\mathbf{x}\mathbf{x}^T W^T W^{-T} \rangle = (I - Q)^{-1} \langle \mathbf{u}\mathbf{u}^T \rangle W^{-T}$. Moreover, if $(I - Q) = \langle \mathbf{u}\mathbf{u}^T \rangle$ holds, $\langle \mathbf{v}\mathbf{x}^T \rangle$ becomes W^{-T}. Therefore, if $(I - Q)$ rapidly converges to $a \langle \mathbf{u}\mathbf{u}^T \rangle$ ($a > 0$), the Linsker rule is equal to the Bell-Sejnowski rule [20]. To achieve such a condition, the learning rule of Q must follow $\tau_Q \dot{Q} = -(Q - (I - a \langle \mathbf{u}\mathbf{u}^T \rangle))$.

To compare the EGHR with the Linsker rule, we consider the case where the sources are not much slower than $\mathbf{v}$ and the sampling time is discrete. If we assume a discrete sampling time Δt and that Q quickly converges to a fixed point, Eq. (5) can be rewritten as

$$\tau_W \dot{W} = \langle a\, \mathbf{v}\, \mathbf{x}^T - g(\mathbf{u})\, \mathbf{x}^T \rangle,$$
$$\tau_v\, (\mathbf{v}(t+\Delta t) - \mathbf{v}) = \Delta t\, (-\mathbf{v} + \mathbf{u} + Q\, \mathbf{v}),$$
$$Q = I - a \langle \mathbf{u}\, \mathbf{u}^T \rangle = I - a\, K\, K^T. \qquad \text{(S41)}$$

From the second and third equations, we obtain a solution of $\mathbf{v}$ as

$$\mathbf{v}(t+\Delta t) - \mathbf{v} = \Delta t/\tau_v\, (\mathbf{u} - a\, K\, K^T\, \mathbf{v}),$$
$$\mathbf{v}(t) = (I - a\, K\, K^T\, \Delta t/\tau_v)\, \mathbf{v}(t-\Delta t) + \Delta t/\tau_v\, \mathbf{u}(t-\Delta t),$$
$$\mathbf{v}(t) = \Delta t/\tau_v \textstyle\sum_{k=0}^{\infty} (I - a\, K\, K^T\, \Delta t/\tau_v)^k\, \mathbf{u}(t-(k+1)\, \Delta t). \qquad \text{(S42)}$$

In order for Eq. (S42) to converge, $|I - aKK^T\Delta t/\tau_v| < 1$ should hold. If K is proportional to a rotation matrix, that is, $K = cR$ ($c > 0$), this condition can be further simplified as $|1 - ac^2\Delta t/\tau_v| < 1$. As ($ac^2\Delta t/\tau_v$) is a positive scalar, we get c's condition for $\mathbf{v}$ to converge as $1 - a\, c^2\, \Delta t/\tau_v > -1$, or equivalently,

$$0 < c < \sqrt{\frac{2\tau_v}{a\Delta t}}. \quad \text{(S43)}$$

This indicates that a large a is likely to make $\mathbf{v}$ unstable if K is started from large initial conditions. The Hebbian term $\langle a\mathbf{v}\mathbf{x}^T\rangle$ in the first equation in Eq. (S41) with a general K can then be calculated as

$$\begin{aligned} a\,\langle \mathbf{v}(t)\,\mathbf{x}(t)^T\rangle &= a\,\Delta t/\tau_v \textstyle\sum_{k=0}^{\infty} (I - a\,K\,K^T \Delta t/\tau_v)^k \langle \mathbf{u}(t-(k+1)\Delta t)\,\mathbf{x}(t)^T\rangle \\ &= a\,\Delta t/\tau_v \textstyle\sum_{k=0}^{\infty} (I - a\,K\,K^T \Delta t/\tau_v)^k K \langle \mathbf{s}(t+(k+1)\Delta t)\,\mathbf{s}(t)^T\rangle K^T W^{-T} \\ &= a\,\Delta t/\tau_v \textstyle\sum_{k=0}^{\infty} (I - a\,K\,K^T \Delta t/\tau_v)^k \rho((k+1)\Delta t)\,K\,K^T W^{-T}. \end{aligned} \quad \text{(S44)}$$

For a strict solution, we need to calculate $\sum_{k=0}^{\infty}(I - \alpha KK^T\Delta t/\tau_v)^k\rho((k+1)\Delta t)$ numerically, where $\rho((k+1)\Delta t) := \langle s_i(t + (k+1)\Delta t)s_i(t)^T\rangle$ is the auto-correlation of a signal train generated from Eq. (7). From Eq. (S44), we define Eq. (17) as the reduced Linsker (R-Linsker) rule. The numerical calculation in Fig. 3B uses this R-Linsker rule to speed up simulations.

Next, we qualitatively explore how the ICA solutions disappear depending on parameter values. Approximating the auto-correlation function by $\rho(t - t') \approx \exp(-|t - t'|/\tau_s)$ for an analytical simplification, we obtain

$$\begin{aligned} a\,\langle \mathbf{v}(t)\,\mathbf{x}(t)^T\rangle &= a\,\Delta t/\tau_v \textstyle\sum_{k=0}^{\infty} (I - a\,K\,K^T \Delta t/\tau_v)^k \exp(-(k+1)\Delta t/\tau_s)\,K\,K^T W^{-T} \\ &= a\,\Delta t/\tau_v \exp(-\Delta t/\tau_s) \textstyle\sum_{k=0}^{\infty} (I - a\,K\,K^T \Delta t/\tau_v)^k \exp(-\Delta t/\tau_s)^k K\,K^T W^{-T} \\ &= a\,\Delta t/\tau_v \exp(-\Delta t/\tau_s)\,\{I - (I - a\,K\,K^T \Delta t/\tau_v)\exp(-\Delta t/\tau_s)\}^{-1} K\,K^T W^{-T} \\ &= \left(\frac{\tau_v(\exp(\Delta t/\tau_s) - 1)}{a\Delta t} K^{-T}K^{-1} + I\right)^{-1} W^{-T}. \end{aligned} \quad \text{(S45)}$$

Therefore, the R-Linsker rule is simplified as

$$\tau_W \dot{W} = \left(\frac{\tau_v(\exp(\Delta t/\tau_s) - 1)}{\alpha\Delta t} K^{-T}K^{-1} + I\right)^{-1} W^{-T} - \langle g(\mathbf{u})\,\mathbf{x}^T\rangle. \quad \text{(S46)}$$

The coefficient matrix of W^{-T} is defined by $C_L(K) := \{\tau_v\,(\exp(\Delta t/\tau_s) - 1)/a\Delta t\; K^{-T} K^{-1} + I\}^{-1}$ (see Table S1). For small Δt, $C_L(K) \approx \{(\tau_v/\tau_s)/a\; K^{-T} K^{-1} + I\}^{-1}$. Indeed, $C_L(K)\,W^{-T}$ dramatically decreases as $(\tau_v/\tau_s)/a$ increases while $\langle g(\mathbf{u})\,\mathbf{x}^T\rangle$ does not. This indicates that the ICA solutions (i.e., equilibrium points) disappear if $(\tau_v/\tau_s)/a$ is too large.

S2.5.2 The Foldiak rule

The Foldiak rule [19] was originally described as shown in Eq. (6). To speed up the

computation to plot the 2-dimensional velocity map in Fig. 3B, we consider a reduced version of the original Foldiak rule, assuming the following conditions: (1) **s**, **x**, **u**, **v** are all two dimensional vectors and the lateral interaction matrix is described by Q=[0, q; q, 0] with $q \le 0$, (2) A is a rotation matrix and W is a rotation matrix up to scaling factor, (3) sources are much more slowly changing than **v**, so that $\mathbf{v} = f_F(\mathbf{u} + Q\mathbf{v} - \mathbf{h})$ holds, (4) h and q converge to a fixed point, so that $\langle v_i \rangle = b$ and $\langle v_i v_j \rangle = b^2$ for $i \neq j$, (5) **s**, **x**, and **u** are symmetric about **0** and **v** is symmetric about $\langle \mathbf{v} \rangle = b\mathbf{1}$, and (6) neuronal nonlinearity $f_F(\mathbf{x})$ is an odd and monotonically increasing nonlinear function up to constant factor b.

We first transform the variables to clarify the symmetry of variables. Let us define mean subtracted output $y_i = v_i - b$ and odd nonlinear function $f(x) = f_F(x) - b$. With this notation the Foldiak rule is described by $\tau_W \dot{W} = a\langle \mathbf{y}\mathbf{x}^T \rangle - W$, where **y** is given by a solution of $y_i = f(u_i + qy_j + qb - h)$ for $i \neq j$, and h and q converge to a fixed point according to $\dot{h} \propto \langle y_i \rangle$ and $\dot{q} \propto -\langle y_1 y_2 \rangle$, so that $\langle y_i \rangle = 0$ and $\langle y_1 y_2 \rangle = 0$ at the fixed point.

For the reasons described below, we can set $h = q = 0$ in this case to simplify the system. Let us start by showing that $h = q = 0$ is a solution of $\langle y_i \rangle = 0$ and $\langle y_1 y_2 \rangle = 0$. The first equation directly follows from $\langle f(u_i) \rangle = 0$ for symmetric **u** and odd function f. To show that $\langle f(u_1) f(u_2) \rangle = 0$, we compute $\langle F(u_1)G(u_2) \rangle = \sum_{n=1}^{4} \langle F(u_1)G(u_2) \rangle_n$ for either odd or even functions F and G. The average $\langle \ \rangle_n$ (n=1, 2, 3, 4) describes the 2-dimensional integration over s_1 and s_2 restricted in the nth quadrant. It is easy to see that

$$\langle F(u_1)G(u_2) \rangle_2 \equiv \int_{-\infty}^{0} ds_1 \int_{0}^{\infty} ds_2 p(s_1)p(s_2)F(c(s_1\cos\theta - s_2\sin\theta))G(c(s_1\sin\theta + s_2\cos\theta))$$

$$= \int_{0}^{\infty} ds_1 \int_{0}^{\infty} ds_2 p(s_1)p(s_2)F(c(-s_1\cos\theta - s_2\sin\theta))G(c(-s_1\sin\theta + s_2\cos\theta))$$

$$= \sigma_F \int_{0}^{\infty} ds_1 \int_{0}^{\infty} ds_2 p(s_1)p(s_2)G(c(s_2\cos\theta - s_1\sin\theta))F(c(s_2\sin\theta + s_1\cos\theta))$$

$$= \sigma_F \langle G(u_1)F(u_2) \rangle_1, \tag{S47}$$

where c is a constant scaling factor, θ is the angle of the rotation by $K = WA$, and σ_F is the parity of F, taking 1 and –1 if F is even and odd function, respectively. Similar calculations for $\langle F(u_1)G(u_2) \rangle_n$ (n = 3 and 4) together yield

$$\langle F(u_1)G(u_2) \rangle = (1 + \sigma_F\sigma_G) \langle F(u_1)G(u_2) \rangle_1 + (\sigma_F + \sigma_G) \langle G(u_1)F(u_2) \rangle_1. \tag{S48}$$

Hence, we find that $\langle f(u_1) f(u_2) \rangle = 0$ by setting $F = G = f$ and $\sigma_f = -1$. This result confirms that $h = q = 0$ is a solution of the Foldiak rule, i.e., $\langle y_i \rangle = 0$ and $\langle y_1 y_2 \rangle = 0$.

We next show that this $h = q = 0$ solution is stable. To demonstrate the linear stability, we express the derivative of the output by $\mathrm{d}y_i = f'(u_i)[f(u_j)\,\mathrm{d}q - b\,\mathrm{d}q - \mathrm{d}h]$ for $i \neq j$, which is evaluated at $h = q = 0$. Using this expression, perturbations of h and q, i.e., dh and dq, develop according to

$$\begin{pmatrix} d\dot{h} \\ d\dot{q} \end{pmatrix} =$$

$$\begin{pmatrix} -\langle f'(u_i)\rangle & \langle f'(u_i)f(u_j) - bf'(u_i)\rangle \\ \langle f'(u_i)f(u_j) + f'(u_j)f(u_i)\rangle & -\langle f'(u_i)f^2(u_j) + f'(u_j)f^2(u_i) - bf'(u_i)f(u_j) - bf'(u_j)f(u_i)\rangle \end{pmatrix}\begin{pmatrix} dh \\ dq \end{pmatrix}$$

$$= \begin{pmatrix} -\langle f'(u_i)\rangle & -b\langle f'(u_i)\rangle \\ 0 & -\langle f'(u_i)f^2(u_j) + f'(u_j)f^2(u_i)\rangle \end{pmatrix}\begin{pmatrix} dh \\ dq \end{pmatrix}. \tag{S49}$$

Note that we used $\langle f'(u_i)f(u_j)\rangle = 0$ for $i \neq j$ in the second line from Eq. (S47). The eigenvalues of the linearized dynamics in Eq. (S49) are $-\langle f'(u_i)\rangle$ and $-\langle f'(u_i)\, f^2(u_j) + f'(u_j)\, f^2(u_i)\rangle$, which are both negative because f is monotonically increasing. Altogether, these results show that $h = q = 0$ is a stable solution.

Finally, we show that h and q must be both zero when the Foldiak rule achieves an ICA solution $W = A^{-1}$. In this case, $u_1 = s_1$ and $u_2 = s_2$ are independent inputs and outputs are given by $y_i = f(s_i + qy_j + qb - h)$. To keep the outputs independent, q must be zero because any none-zero q would introduce some dependency between the two outputs. If $q = 0$, then, $h = 0$ is the unique solution to $\langle y_i\rangle = 0$ for monotonically increasing f.

Altogether, $h = q = 0$ is a stable solution of the Foldiak rule throughout the learning in this case, and $h = q = 0$ must hold when it eventually achieves the ICA solution. Although it is possible to initialize h and q to a non-zero value, the output **y** becomes multi-stable if $|q|$ is large. Hence, it is most natural to initially set $h = q = 0$, following the original proposal [19]. Accordingly, Eq. (6) can be simplified as Eq. (18). We define Eq. (18) to be the reduced Foldiak (R-Foldiak) rule. The numerical calculation in Fig. 3B is based on this R-Foldiak rule.

S2.6 Equilibrium points of conventional ICA rules

S2.6.1 The Bell-Sejnowski, Amari, and Cichocki rules

Let us show that $W = A^{-1}$ is an equilibrium point of the Bell-Sejnowski, Amari, and Cichocki rules based on a previous study [32]. We can confirm this by checking $\dot{W}$ after

substituting $W = A^{-1}$ into each equation. Let us start checking this for the Bell-Sejnowski rule. From Eq. (2), an equilibrium state of W is given by

$$W^{-T} = \langle g(\mathbf{u})\,\mathbf{x}^T\rangle_{p(\mathbf{x})}. \tag{S50}$$

If $W = A^{-1}$, $\mathbf{u} = W\mathbf{x}$ becomes $\mathbf{u} = \mathbf{s}$ and $\langle g(\mathbf{u})\mathbf{x}^T\rangle_{p(\mathbf{x})}$ becomes $\langle g(\mathbf{s})\mathbf{s}^T\rangle_{p0(\mathbf{s})}\,A^T$. Note that $\langle g(\mathbf{s})\mathbf{s}^T\rangle_{p0(\mathbf{s})}$ is a diagonal matrix since $s_1, \ldots, s_N$ are independent of each other, and its diagonal elements are one, i.e., $\langle g(\mathbf{s})\mathbf{s}^T\rangle_{p0(\mathbf{s})} = I$ (see S2.1.1). As $W = A^{-1}$ satisfies Eq. (S50), $W = A^{-1}$ is one of the solutions to the Bell-Sejnowski rule.

The equilibrium points of the Amari and Cichocki rules are calculated similarly. The difference among the Bell-Sejnowski, Cichocki, and Amari rules is only the multiplication of some regular matrix from the right, which does not remove an equilibrium point. Therefore, $W = A^{-1}$ is also an equilibrium state of the Amari and Cichocki rules.

S2.6.2 The Linsker rule

As shown in S2.5.1, the Linsker rule becomes equal to the Bell-Sejnowski rule when the input is much slower than the dynamics of $\mathbf{v}$ [20]; therefore, $W = A^{-1}$ is also an equilibrium state of the Linsker rule. However, when sources obey a rapidly changing super-Gaussian distribution (e.g., a Laplace distribution), the Linsker model does not always have a stable equilibrium point depending on the time constant and distribution shape of the source. Indeed, the existence of ICA solutions for the R-Linsker rule (Eq. (S40)) depends on the parameters when the sources are not much slower than $\mathbf{v}$. If we assume $K = c\,I$ ($c > 0$), the second term on the right of Eq. (S46) becomes $\langle g(\mathbf{u})\mathbf{x}^T\rangle = \langle g(c\mathbf{s})\mathbf{s}^T\rangle A^T$. If we assume sources obey a Laplace distribution, we obtain $\langle g(c\mathbf{s})\mathbf{s}^T\rangle = \langle \mathrm{sgn}(\mathbf{s})\mathbf{s}^T\rangle = I$, which is independent of c. The fixed point is then calculated as

$$\left(\frac{\tau_v(\exp(\Delta t/\tau_s)-1)}{c^2 a\Delta t}+1\right)^{-1}\frac{1}{c} = 1,$$

$$c^2 - c + \frac{\tau_v(\exp(\Delta t/\tau_s)-1)}{a\Delta t} = 0,$$

$$c = \frac{1}{2}\left(1 \pm \sqrt{1-\frac{4\tau_v(\exp(\Delta t/\tau_s)-1)}{a\Delta t}}\right). \tag{S51}$$

Therefore, in order for K to converge to a non-zero finite value, the radial distance c should be started from

$$\frac{1}{2}\left(1-\sqrt{1-\frac{4\tau_v(\exp(\Delta t/\tau_s)-1)}{a\Delta t}}\right) < c < \sqrt{\frac{2\tau_v}{a\Delta t}}, \tag{S52}$$

and the fixed point of c is

$$c = \frac{1}{2}\left(1+\sqrt{1-\frac{4\tau_v(\exp(\Delta t/\tau_s)-1)}{a\Delta t}}\right). \tag{S53}$$

Accordingly, in order for the fixed point to exist, $\tau_v/a\Delta t$ should be

$$\tau_v/a\Delta t < \frac{1}{4(\exp(\Delta t/\tau_s)-1)}. \tag{S54}$$

Note that a also controls the stability of the dynamics of $\mathbf{v}$. If a is set too large, the dynamics of $\mathbf{v}$ does not converge.

S2.6.3 The Foldiak rule

The Foldiak rule (Eq. (6)) can perform ICA only when A is a rotation matrix. To calculate the Foldiak rule's equilibrium point, we assume that $W = A^{-1}$ holds and that $\dot{Q}$ and $\dot{\mathbf{h}}$ have converged to the zero fixed point (see S2.5.2). From $\mathbf{h} = \mathbf{0}$, the output satisfies $\langle \mathbf{v} \rangle = b\mathbf{1}$. Thus, the last equation in Eq. (6) becomes $\tau_W \dot{W} = a\langle \mathbf{v}\mathbf{x}^T \rangle - W$. If $W = A^{-1}$, the output is given by $\mathbf{v} = f_F(\mathbf{s})$. Therefore, $\langle \mathbf{v}\mathbf{s}^T \rangle$ must be proportional to an identical matrix. In this case, the equilibrium condition is given by $0 = \dot{W} = a\langle \mathbf{v}\mathbf{s}^T \rangle A^T - A^{-1}$, or equivalently, $A^{-1}A^{-T} = a\langle \mathbf{v}\mathbf{s}^T \rangle \propto I$. Accordingly, in order for the Foldiak rule to achieve ICA, A must be proportional to a rotation matrix. Moreover, if a is chosen so that $a\langle f_F(s_i)s_i \rangle = 1$, A^{-1} should be equal to A^T, i.e., A must be a rotation matrix in order for the Foldiak rule to have the ICA solution.

S2.7 Linear stability of conventional ICA rules

S2.7.1 The Bell-Sejnowski, Amari, and Linsker rules

Here, we repeat the linear stability analysis of the the Bell-Sejnowski, Amari, and Linsker rules [32] to apply it to the Cichocki and Foldiak rules in the following sections. Let us define K as $K = WA$ and represent $\mathbf{u}$ as $\mathbf{u} = K\mathbf{s}$. Linear stability is confirmed by showing that when $K = WA = I + \varepsilon J$ is substituted into each ICA rule, where positive constant ε is small enough, the signs of the elements of $\dot{K}$ never change, regardless of the value of J. Specifically, the Amari rule (Eq. (3)) is rewritten as $\dot{K} \propto \langle I - g(K\mathbf{s})\mathbf{s}^T K^T \rangle K$ (see Table S1). If we assume that W is near to A^{-1} as $K = I + \varepsilon J$, ($\varepsilon << 1$), the Taylor expansion of $g(\mathbf{s} + \varepsilon J\, \mathbf{s})$ is given by $g(\mathbf{s} + \varepsilon J\mathbf{s}) = g(\mathbf{s}) + \Lambda\varepsilon J\, \mathbf{s} + O(|\varepsilon J\, \mathbf{s}|^2)$, where Λ =

Diag[$g'(\mathbf{s})$] is a diagonal matrix of $g'(\mathbf{s})$. Using the relationship of $\langle g(\mathbf{s})\mathbf{s}^T\rangle = I$, Eq. (3) is further calculated as

$$\begin{aligned}\varepsilon \dot{J} &\propto \langle I - g(\mathbf{s} + \varepsilon J \mathbf{s})\, \mathbf{s}^T (I + \varepsilon J)^T\rangle\, (I + \varepsilon J) \\ &\approx \langle I - (g(\mathbf{s}) + \Lambda\, \varepsilon J \mathbf{s})\, \mathbf{s}^T (I + \varepsilon J^T)\rangle\, (I + \varepsilon J) \\ &\approx \langle I - (g(\mathbf{s})\, \mathbf{s}^T + \Lambda\, \varepsilon J \mathbf{s}\, \mathbf{s}^T + g(\mathbf{s})\, \mathbf{s}^T \varepsilon J^T)\rangle\, (I + \varepsilon J) \\ &\approx -\varepsilon \langle \Lambda J \mathbf{s}\, \mathbf{s}^T + g(\mathbf{s})\, \mathbf{s}^T J^T\rangle \\ &= -\varepsilon\, (\langle \Lambda J \mathbf{s}\, \mathbf{s}^T\rangle + J^T). \end{aligned} \tag{S55}$$

Because the (i, j)th element of $\langle \Lambda J\mathbf{s}\mathbf{s}^T\rangle$ is calculated as $\langle \Lambda J\mathbf{s}\mathbf{s}^T\rangle_{ij} = \langle \sum_k (\Lambda J)_{ik} s_k s_j\rangle = \sum_k \langle \Lambda_{ii} J_{ik} s_k s_j\rangle = \langle \Lambda_{ii} J_{ij} s_j^2\rangle = \langle g'(s_i) s_j^2\rangle J_{ij}$, the (i, j)th element of Eq. (S55) is calculated as

$$\dot{J}_{ij} \propto -\langle g'(s_i)\, s_j^2\rangle\, J_{ij} - J_{ji}. \tag{S56}$$

When $i = j$, Eq. (S56) is calculated as $\dot{J}_{ii} \propto -(\langle g'(s_i)s_i^2\rangle + 1)\, J_{ii}$; therefore, when $\langle g'(s_i)s_i^2\rangle + 1 > 0$ holds, J_{ii} converges to zero. When $i \neq j$, Eq. (S56) is calculated as

$$\dot{J}_{ij} \propto -\langle g'(s_i)\rangle\, \langle s_i^2\rangle\, J_{ij} - J_{ji}\,. \tag{S57}$$

From Eq. (S57), the relationship of $\dot{J}_{ji} = -\langle g'(s_i)\rangle\langle s_i^2\rangle J_{ji} - J_{ij}$ also holds; therefore, we obtain

$$\begin{aligned}\dot{J}_{ij} + \dot{J}_{ji} &\propto -(\langle g'(s_i)\rangle\, \langle s_i^2\rangle + 1)\, (J_{ij} + J_{ji}), \\ \dot{J}_{ij} - \dot{J}_{ji} &\propto -(\langle g'(s_i)\rangle\, \langle s_i^2\rangle - 1)\, (J_{ij} - J_{ji}). \end{aligned} \tag{S58}$$

When $\langle g'(s_i)\rangle\langle s_i^2\rangle > 1$, $\dot{J}_{ij} + \dot{J}_{ji}$ and $\dot{J}_{ij} - \dot{J}_{ji}$ converge to zero with increasing t, i.e., J_{ij} and J_{ji} converge to zero. Because $s_1, \ldots, s_N$ independently obey an identical distribution, the necessary and sufficient conditions for J to converge are $\langle g'(s_i)s_i^2\rangle + 1 > 0$ and $\langle g'(s_i)\rangle\langle s_i^2\rangle > 1$. In this condition, $W = A^{-1}$ is a stable equilibrium point of the Amari rule. The condition is the same as that studied by Amari using the second differential form of the cost function L_A [32].

Moreover, the Bell-Sejnowski rule is represented as a multiplication of the Amari rule by a positive definite matrix $W^{-1}W^{-T}$ from the right (for any matrix W, $W^{-1}W^{-T}$ is a positive definite matrix); therefore, the conditions where $W = A^{-1}$ is a stable equilibrium point of the Bell-Sejnowski rule is the same as that of the Amari rule. If the sources are sufficiently slow, the Linsker rule converges into the Bell-Sejnowski rule and is therefore linearly stable for the same conditions.

S2.7.2 The Cichocki rule

Similarly, the linear stability of the Cichocki rule at $W = A^{-1}$ is calculated. Eq. (4) is rewritten as

$$\begin{aligned} \dot{K} &\propto \langle I - g(K\mathbf{s})\,\mathbf{s}^T K^T\rangle A \\ &\approx -\varepsilon\,(\langle \Lambda\, J\,\mathbf{s}\,\mathbf{s}^T\rangle + J^T)\,A. \end{aligned} \tag{S59}$$

By comparing Eq. (S59) with Eq. (S55), the condition of linear stability depends on the eigenvalues of A and the initial condition of W. The Cichocki rule is stable at some ICA solutions if some of eigenvalues of A are positive, but its performance depends on the initial condition of W. In contrast, the Cichocki rule is stable at the all ICA solutions if all eigenvalues of A are non-negative and the sum of all eigenvalues is positive.

S2.7.3 The Foldiak rule

We assume the same assumptions with S2.5.2. Thus, the Foldiak rule is equal to Eq. (18) when $W = A^{-1}$ and Eq. (18) is a good approximation of the original Foldiak rule when W is closed to A^{-1}. Using $K = WA$, Eq. (18) is calculated as

$$\dot{K} \propto a\langle f_F(K\mathbf{s})\mathbf{s}^T A^T A\rangle - K. \tag{S60}$$

Transform matrix A is required to be a rotation matrix in order for the Foldiak model to achieve an equilibrium state at $W = A^{-1}$ (see S2.6.3), so that $A^T A = I$. By substituting $K = I + \varepsilon J$ into Eq. (S60), we obtain

$$\begin{aligned} \varepsilon\,\dot{J} &\propto a\,\langle f_F(\mathbf{s} + \varepsilon J\,\mathbf{s})\,\mathbf{s}^T\rangle - (I + \varepsilon\,J) \\ &\approx a\,\langle (f(\mathbf{s}) + \Lambda\,\varepsilon J\,\mathbf{s})\,\mathbf{s}^T\rangle - (I + \varepsilon\,J) \\ &= a\,\langle f(\mathbf{s})\,\mathbf{s}^T + \Lambda\,\varepsilon J\,\mathbf{s}\,\mathbf{s}^T\rangle - (I + \varepsilon\,J) \\ &= a\,(\langle f(s_i)\,s_i\rangle\,I + \langle \Lambda\,\varepsilon\,J\,\mathbf{s}\,\mathbf{s}^T\rangle) - (I + \varepsilon\,J) \\ &= \varepsilon\,a\,\langle \Lambda\,J\,\mathbf{s}\,\mathbf{s}^T\rangle - \varepsilon\,J, \end{aligned} \tag{S61}$$

where $\Lambda = \mathrm{Diag}[f_F'(s_i)]$. The (i, j)th element of $\dot{J}$ is calculated as

$$\begin{aligned} \dot{J}_{ij} &\propto (a\,\langle \Lambda\,J\,\mathbf{s}\,\mathbf{s}^T\rangle_{ij} - J_{ij}) \\ &= (a\,\langle \textstyle\sum_k (\Lambda\,J)_{ik}\,s_k\,s_j\rangle - J_{ij}) \\ &= (a\,\textstyle\sum_k J_{ik}\,\langle \Lambda_{ii}\,s_k\,s_j\rangle - J_{ij}) \end{aligned}$$

$= (a\, J_{ij}\, \langle A_{ii}\, s_j^2 \rangle - J_{ij})$

$= (a\, \langle A_{ii}\, s_j^2 \rangle - 1)\, J_{ij}.$ (S62)

When $a\langle A_{ii}\, s_i^2 \rangle - 1 < 0$ and $a\langle A_{ii} \rangle \langle s_i^2 \rangle - 1 < 0$ hold, J_{ij} converges to zero. Assuming $f_F(s_i)$ is a sigmoid function $f_F(s_i)$ = sig(γs_i) and γ is large, $\langle A_{ii}\, s_i^2 \rangle$ becomes zero and $\langle A_{ii} \rangle \langle s_i^2 \rangle$ becomes $2\langle \delta(s_i) \rangle \langle s_i^2 \rangle = 2p_0(0)\langle s_i^2 \rangle$ since $\langle \delta(s_i) \rangle = \int \delta(s_i) p_0(s_i) ds_i = p_0(0)$. Therefore, if and only if $ap_0(0)\langle s_i^2 \rangle$ is smaller than one, the Foldiak model is linearly stable. For example, when a source obeys a normalized uniform distribution, $p_0(0)$ and $\langle |s_i| \rangle$ are respectively calculated as $p_0(0) = 1/2\sqrt{3}$ and $\langle |s_i| \rangle = 2\cdot(\sqrt{3})^2/2\cdot(1/2\sqrt{3}) = \sqrt{3}/2$, so that, since $p_0(0)\langle s_i^2 \rangle / \langle |s_i| \rangle = 1/3$, J_{ij} converges to zero. When a source obeys a normalized Gaussian distribution, $p_0(0)$ and $\langle |s_i| \rangle$ are respectively calculated as $p_0(0) = 1/\sqrt{2\pi}$ and $\langle |s_i| \rangle = 2/\sqrt{2\pi} \int_0^\infty s_i \exp(-s_i^2/2)\, ds_i = 2/\sqrt{2\pi}\, [-\exp(-s_i^2/2)]_0^\infty = 2/\sqrt{2\pi}$, so that, since $p_0(0)\langle s_i^2 \rangle / \langle |s_i| \rangle = 1/2$, $\dot{J}_{ij}$ is always equal to zero. When a source obeys a normalized Laplace distribution ($p_0(s_i) = 1/\sqrt{2}\, \exp(-\sqrt{2}\,|s_i|)$), if we use $f_F(s_i) = 4.444/(1+\exp(-\sqrt{2}\, s_i^3))$, $p_0(0)\langle s_i^2 \rangle / \langle |s_i| \rangle$ is calculated as $p_0(0)\, \langle s_i^2 \rangle / \langle |s_i| \rangle = -0.9447\ldots$; therefore, J_{ij} converges to zero.

S2.8 Performance of the conventional ICA rules in the undercomplete condition

In contrast to the EGHR, conventional ICA rules do not straightforwardly work in the undercomplete condition (Fig. 5). Unlike the EGHR rule (see Methods), the optimal representation $K = (I, I)^T$ is not an equilibrium point of the Amari rule because when we substitute $K = (I, I)^T$ into the Amari rule, $\dot{K}$ is rewritten as $\dot{K} \propto \langle I - g(K\mathbf{s})(K\mathbf{s})^T \rangle\, K \neq 0$; hence, $K = (I, I)^T$ never becomes an equilibrium point of the Amari rule. Moreover, $K = (I, 0)^T$ is not an equilibrium point of the Amari rule either. For the same reason, $K = (I, I)^T$ and $(I, 0)^T$ are not equilibrium points of the Bell-Sejnowski, Cichocki, or Linsker rules. Importantly, inputs are definitely correlated in the undercomplete condition. Because the Foldiak rule requires A to be a rotation and scaling matrix, it does not have ICA solutions in the undercomplete condition either.
